# Supplementary material for: Chiral plasmonic superlattices from template-assisted assembly of achiral nanoparticles
Source: Nat Commun. 2025 Feb 16;16:1687. doi: 10.1038/s41467-025-56999-0 (PMC11830766; doi:10.1038/s41467-025-56999-0)
Supplement: Supplementary file 1 — Supplementary Information [file 41467_2025_56999_MOESM1_ESM.pdf]

Supplementary information for

Chiral plasmonic superlattices from template-assisted assembly of achiral nanoparticles

Xiaoyu Qi[1], Luis Alberto Pérez[1]*, Jose Mendoza-Carreño[1], Miquel Garriga[1], Maria Isabel Alonso[1], Agustín Mihi[1]*

Affiliations:

[1]Institute of Materials Science of Barcelona ICMAB-CSIC; Campus UAB, Bellaterra, 08193, Spain.

*Corresponding author. Emails: lperez@icmab.es, amihi@icmab.es

Suppl. Note 1. Design and numerical calculations of triskelion pattern

For the type of 2D lattice that should be used, both hexagonal and square lattices were examined. Symmetry considerations indicated that the hexagonal lattice preserves the threefold symmetry of the pattern. Simulation results further confirmed that the hexagonal lattice pattern produces higher CD and lower LD than a similar square lattice pattern.

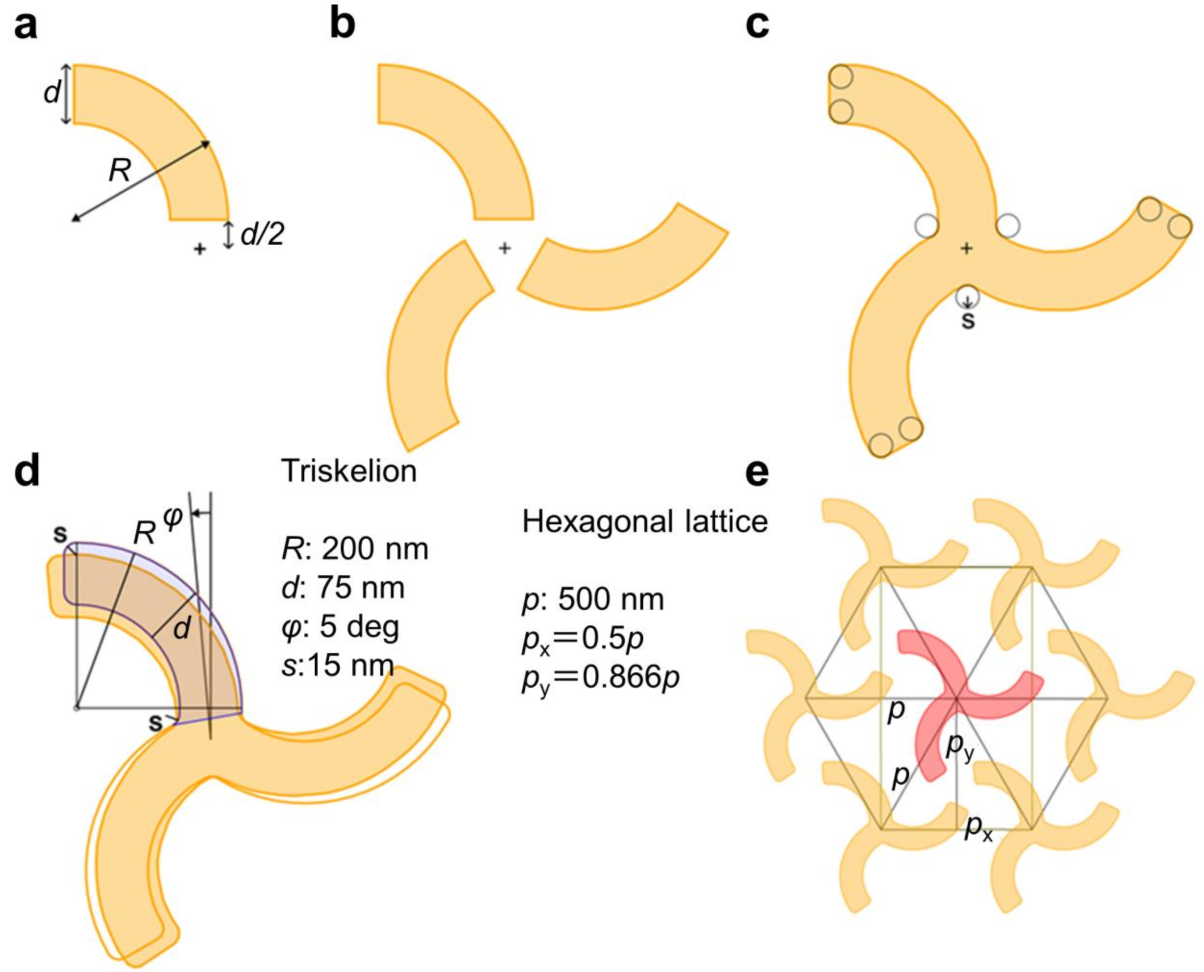

Supplementary Fig. 1. Geometry scheme used to define the L-triskelion polygon shape. (**a**) Starting with a quarter ring of radii *R* and (*R*-*d*), two replicas are obtained by rotating the original by 2π/3 and 4π/3, respectively. (**b**) Tangential circles of radius *s* are used to connect the three polygons and to smooth sharp corners. (**c**) The final polygon is obtained by rotating the triskelion at an angle *φ*. Shape for the corresponding R-triskelion is obtained by changing the sign of vertices' x coordinate. (**d**) The pattern was obtained by placing a triskelion in the unit cell of a 2D hexagonal lattice, an equilateral triangle (side p). For FDTD simulations a rectangular lattice (p_x, $2p_y$) with two triskelia per unit cell is used.

Numerical Modeling of Single Triskelion Structures

The optical response of an isolated triskelion structure and triskelion arrays was investigated using numerical simulations based on the FDTD method. The simulations were conducted within the commercial software Ansys FDTD-Solutions (Lumerical). The modeled geometry consisted of a

single triskelion or triskelion arrays (Supplementary Fig. 1) situated on a glass substrate. Right-handed circular polarization (RCP) and left-handed circular polarization (LCP) light illumination was simulated by launching two co-propagating light sources with orthogonal polarizations and a phase difference of $\pm\pi/2$ using planes waves or the total-field scattered-field (TFSF) source in Ansys-Lumerical. Perfectly matched layers (PMLs) were implemented on all simulation boundaries to absorb outgoing light waves and minimize back-reflections that could interfere with the results.

The PML was placed at least half the maximum wavelength of interest, ensuring efficient light absorption and minimizing spurious reflections within the simulation volume. For the analysis of electric-field distribution within the triskelion, a monitor was strategically positioned at mid-height within the triskelion plane, affording a comprehensive cross-sectional depiction of electric field electric-field intensity. Triskelion target compose of nanoparticles was developed by setting close-packed 27 nm Au NPs, guided by the triskelion's coordinates as a boundary condition. To model the capping of each nanoparticle, a dielectric shell with a refractive index of 1.5 and a thickness of 2 nm was employed.

Supplementary Fig. 2 presents the calculated transmittance, CD and LD spectra for two distinct structures: triskelion arrays constructed from bulk material with dimensions as described in Supplementary Fig. 1 and a height of 100 nm (Supplementary Fig. 2a) and close-packed 27 nm Au NPs with a 2 nm capping layer (Supplementary Fig. 2b). The LD spectra were obtained by considering the difference in light extinction of the structures when irradiated with linearly polarized light along the *x* and *y* directions (see Supplementary Fig. 1). Similarly, the CD spectra were calculated based on the difference in extinction between the two circularly polarized components of light.

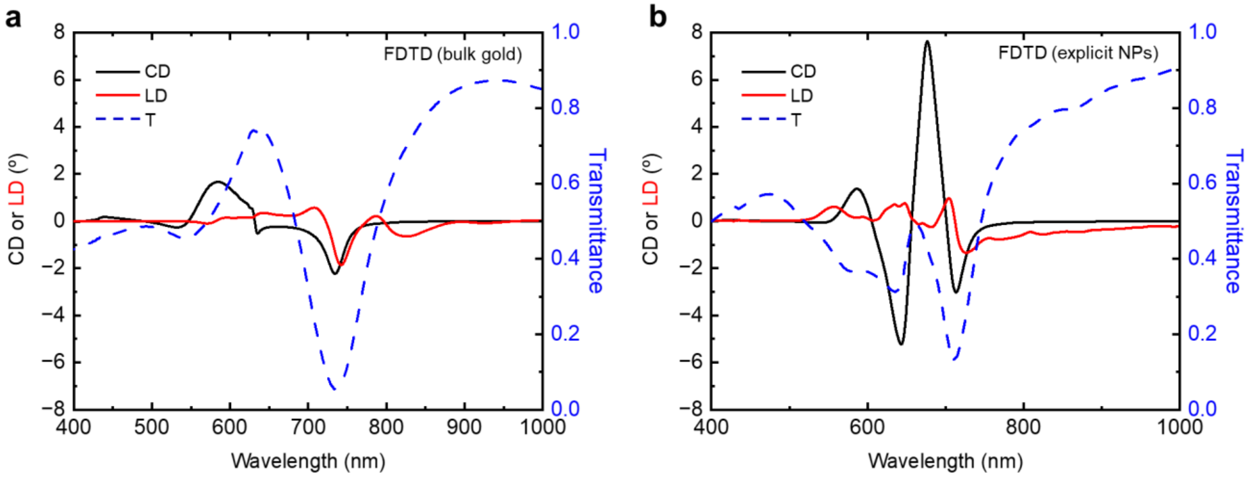

Supplementary Fig. 2. **Calculated transmittance, circular dichroism and linear dichroism for triskelion arrays**. Triskelions compose of (**a**) bulk gold and (**b**) 27 nm gold nanoparticles.

To elucidate the mechanism underlying the chiral behavior of triskelion arrays, we computationally modeled the optical properties of isolated triskelion and triskelion lattices. Supplementary Fig. 3a presents the extinction coefficients of a single triskelion under circularly polarized illumination. A pronounced extinction maximum is observed near 600 nm for both circular handedness, attributable to the LSPR of the triskelion shape arising from the collective response of the near-field coupled constituent nanoparticles. The observed differential optical response confirms the chiral nature of the triskelion. The *g*-factor, as depicted in Supplementary

Fig. 3c, exhibits a chiral response profile within the 600-700 nm range. Furthermore, Supplementary Fig. 3b displays the transmission spectra of hexagonal triskelion arrays with a period of 500 nm for both circular polarization handedness. The dashed red dashed vertical line indicates the Rayleigh Anomaly (RA) of the array, at that wavelength a significant difference in transmittance between the two polarizations is observed. This finding is corroborated by the calculated *g*-factor for the arrays, which reveals an enhanced chiral response compared to an isolated triskelion (Supplementary Fig. 3c, red line). These results suggest that the hybridization of the chiral LSPR mode of each triskelion with the lattice diffraction mode, leads to a SLR with a strong chiral character, becoming the dominant mechanism responsible for the amplified chiral response in triskelion arrays.

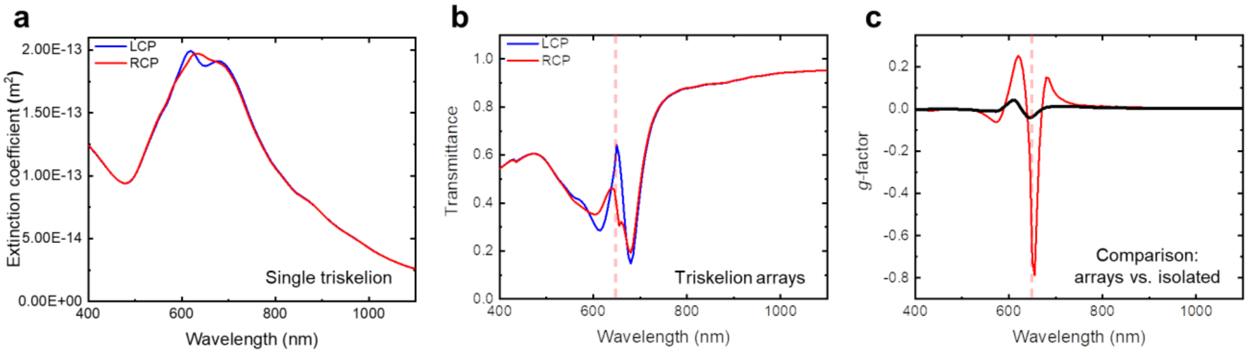

Supplementary Fig. 3. Optical properties modelling. FDTD simulation of (**a**) the extinction coefficient in squared meters of an isolated triskelion, (**b**) transmittance spectra of triskelions arrays under circularly polarized light. (**c**) Compared *g*-factor response of single triskelions (black) versus triskelion arrays (red). LP = 500 nm, Au NPs 27 nm, interparticle distance 6 nm.

Suppl. Note 2. Synthesis of the different colloids

Supplementary Table 1. Details of the growth steps for the synthesis of silver nanospheres.

Diameter	Growing steps	Re-growing steps	Relative centrifugal force (*g*)
20 nm	No growing steps	No re-growing steps	16099×
27 nm	200 µL of 25 mM SC,<br>500 µL of 2.5 mM TA,<br>and 500 µL of 25 mM $AgNO_3$<br>(reaction time: 15 min)	No re-growing steps	12298×
32 nm	1 mL of 25 mM SC,<br>3 mL of 2.5 mM TA,<br>and 2 mL of 25 mM $AgNO_3$<br>(reaction time: 30 min)	No re-growing steps	9056×
36 nm	1 mL of 25 mM SC,<br>3 mL of 2.5 mM TA,<br>and 2 mL of 25 mM $AgNO_3$<br>(reaction time: 30 min)	1 mL of 25 mM SC,<br>3 mL of 2.5 mM TA,<br>and 2 mL of 25 mM $AgNO_3$<br>(reaction time: 30 min)	7155×

Supplementary Table 2. Details of the growth and etching steps related to the synthesis process of gold nanospheres.

Diameter (nm)	Volume of 10 nm seeds (μL)	Oxidative etching	Etching time (min)	Relative centrifugal force (*g*)
27	3500	50 μL of NaClO	20	16099×
45	1000	50 μL of NaClO + 10 μL of $HAuCl_4$	40	7155×
54	700	50 μL of NaClO + 12.5 μL of $HAuCl_4$	50	4724×
70	500	50 μL of NaClO + 20 μL of $HAuCl_4$	60	2795×

Suppl. Note 3. Characterization and concentration calculation of colloidal solutions

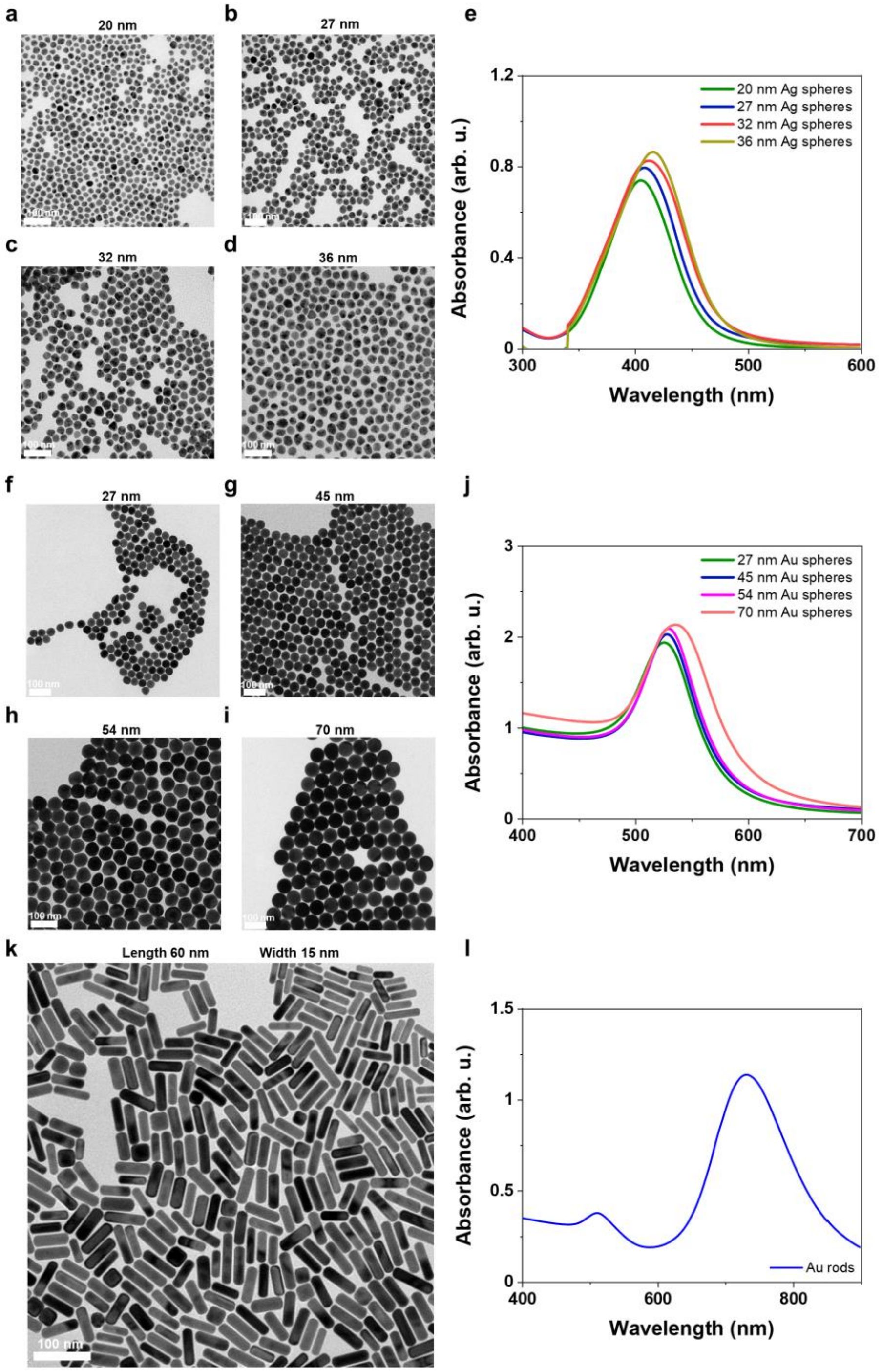

Supplementary Fig. 4. (**a-d**) **Metal colloids characterization.** TEM images and (**e**) UV-Vis extinction spectra of silver nanospheres with different sizes. (**f-i**) TEM images and (**j**) UV-Vis extinction spectra of gold nanospheres with different sizes. (**k**) TEM image and (**l**) UV-Vis extinction spectrum of gold nanorods. Source data are provided as a Source Data file.

Supplementary Table 3. Parameters used to calculate the concentration of Ag nanoparticle solutions for each synthesized size.

Diameter (nm)	ε (10^8 M^{-1} cm^{-1})
20	41.8
27	101*
32	181
36	255

*this value was obtained by interpolation from the tabulated data in ref. 56 main text

For example, in the case of 20 nm silver nanospheres, the estimated number of Ag atom per NP was calculated as following: the volume of each 20 nm silver nanosphere is 4.2×10^{-18} cm^3:

$$\frac{4.2 \times 10^{-18}\ \text{cm}^3\ \text{NP}^{-1} \times 10.49\ \text{g cm}^{-3} \times\ 6.022 \times 10^{23} \text{Ag}^0\ \text{atoms mol}^{-1}}{107.87\ \text{g mol}^{-1}} \quad (1)$$

$$= 2.45 \times 10^5\ \text{Ag}^0\ \text{atoms NP}^{-1}$$

When the stock dispersion is diluted 20,000 times, the absorbance value at λ_{max} was 0.56. According to the Beer-Lambert law, the concentration of Ag NPs is

$$\frac{0.56}{41.8 \times 10^8 \text{M}^{-1}\ \text{cm}^{-1} \times 1\ \text{cm}} \times 20{,}000 = 2.68 \times 10^{-6}\ \text{mol L}^{-1} \quad (2)$$

Finally, the concentration of Ag^0 in the stock dispersion is determined as

$$2.45 \times 10^5\ \text{Ag}^0\ \text{atoms NP}^{-1} \times 2.68 \times 10^{-6}\ \text{mol L}^{-1} = 0.657\ \text{mol L}^{-1}\ (657\ \text{mM}) \quad (3)$$

The silver nanospheres were also dispersed in a mixture of H_2O: ethanol (3:2) with Ag concentration ($[Ag^0]$) of 15, 30 and 50 mM with a final concentration of CTAC of 50 μM.

For example, in the case of 27 nm gold nanospheres. After 1,000 times dilution of the gold stock solution, UV-Vis was measured. The value of 400 nm of this diluted solution was 0.68, so the concentration of Au^0 of stock dispersion is

$$\frac{0.68 \times 1.2}{0.5} \times 1{,}000 = 1632\ \text{mM} \quad (4)$$

The gold nanospheres/nanorods were dispersed in the mixture of H_2O: ethanol (3:2), with gold concentration ($[Au^0]$) = 15, 30 and 50 mM with a final concentration of CTAC of 50 μM.

Suppl. Note 4. Photographs of the samples

Photographs of some of the samples fabricated with the templated-assembly are shown in Supplementary Fig. 5 below. Samples produced from silver and gold nanospheres were very uniform, along the triskelia patterns region (16 mm^2 square). The samples showed iridescence in the ordered pattern, with a metallic frame around corresponding to randomly organized colloids.

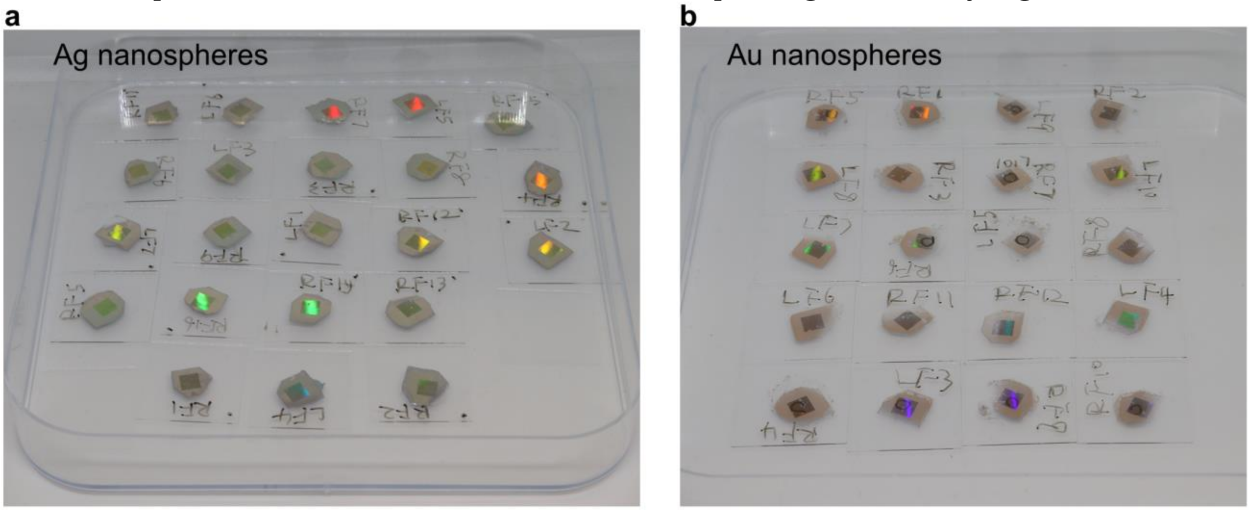

Supplementary Fig. 5. Photographs of set of samples. (**a**) 32 nm silver nanospheres, (**b**) 45 nm gold nanospheres.

Suppl. Note 5. Electron microscopy characterization of triskelion arrays

TEM

Initial gold and silver nanoparticles TEM images were collected with a JEOL 1210 TEM instrument operating at 120 kV. For a comprehensive analysis, STEM and HRTEM were employed to characterize the triskelion arrays. The arrays were assembled directly onto Formvar Carbon support film on copper 200 mesh TEM grids. The assembly procedure mirrored that used for other substrates. Briefly, 1 µL of the 27 nm Au NP colloidal dispersion was deposited onto a PDMS stamp and immediately covered with the TEM grid. The solvent was then allowed to evaporate, followed by the removal of the TEM grid with the assembled structures.

Supplementary Fig. 6 displays the resulting (S)TEM images of the assemblies. The morphology of each triskelion is clearly resolved, with no significant lattice defects observed, as confirmed by the low-magnification STEM image in Supplementary Fig. 6a.

Panels c and d of Supplementary Fig. 6 present HRTEM images acquired using the FEI Tecnai G2 F20 HRTEM microscope operated at 120 kV. The inset in Supplementary Fig. 6d highlights the 0.236 nm spacing, corresponding to the (111) crystal plane of one of the assembled Au nanoparticles.

EELS

Acquisition of EELS spectra and maps was conducted using a Spectra 300 (60-300 kV) monochromated (scanning)-transmission electron microscope with double aberration correction (ThermoFisher Scientific) equipped with a Gatan Continuum K3 EELS spectrometer featuring

direct electron detection. The microscope was operated at 120 kV and the zero-loss peak (ZLP) was optimized using the X-FEG/UltiMono.

The inset in Supplementary Fig. 6a indicates the region where EELS characterization was conducted (Fig. 1e). Supplementary Fig. 6b shows the integrated EELS spectrum for the designated region. A spatial resolution of 10 nm was employed for the mapping shown in Fig. 1d.

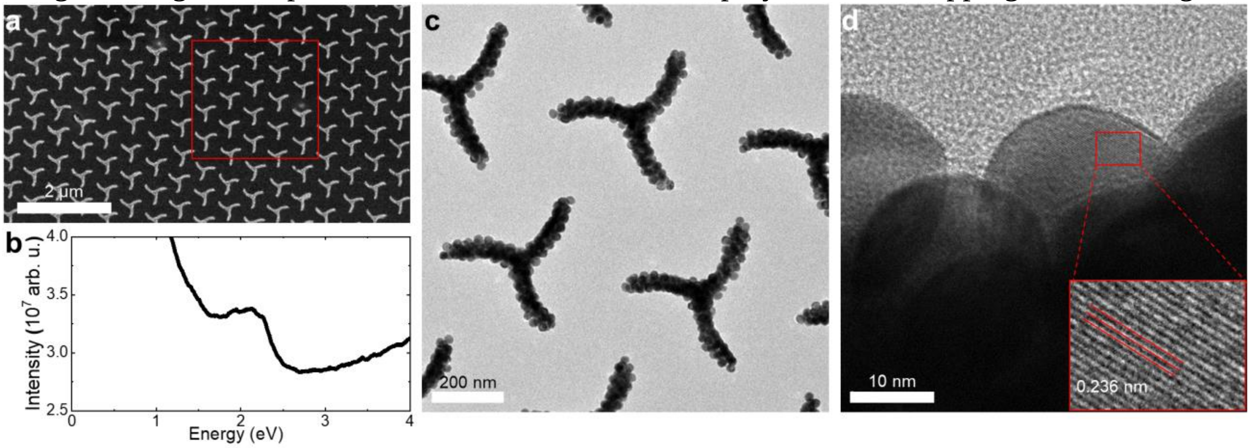

Supplementary Fig. 6. Electron microscopy images and EELS spectra from a triskelia array (27 nm Au NPs). (**a**) Low-magnification STEM image of the triskelia array. The red square indicates the region analyzed using EELS. (**b**) The corresponding spectrum represents the integrated signal from this region. (**c-d**) HRTEM images of the triskelia array.

SEM

The morphology of the triskelion arrays was directly assessed using SEM on the glass-supported structures using FEI QUANTA 200 Field Emission Gun and FEI Magellan 400L microscopes.

Arrays with silver nanospheres. Samples fabricated with different sizes of Ag colloids (20, 27, 32 and 36 nm) using dispersions with [Ag^0] of 50 mM were characterized by SEM (Supplementary Figs. 7-9). Low-magnification images demonstrate the uniformity and long range order of the assembled patterns, with low level of defects. High magnification images clearly show the well-defined triskelion shape with most silver nanospheres located in the triskelion motif, with only a small number of colloids outside the triskelia.

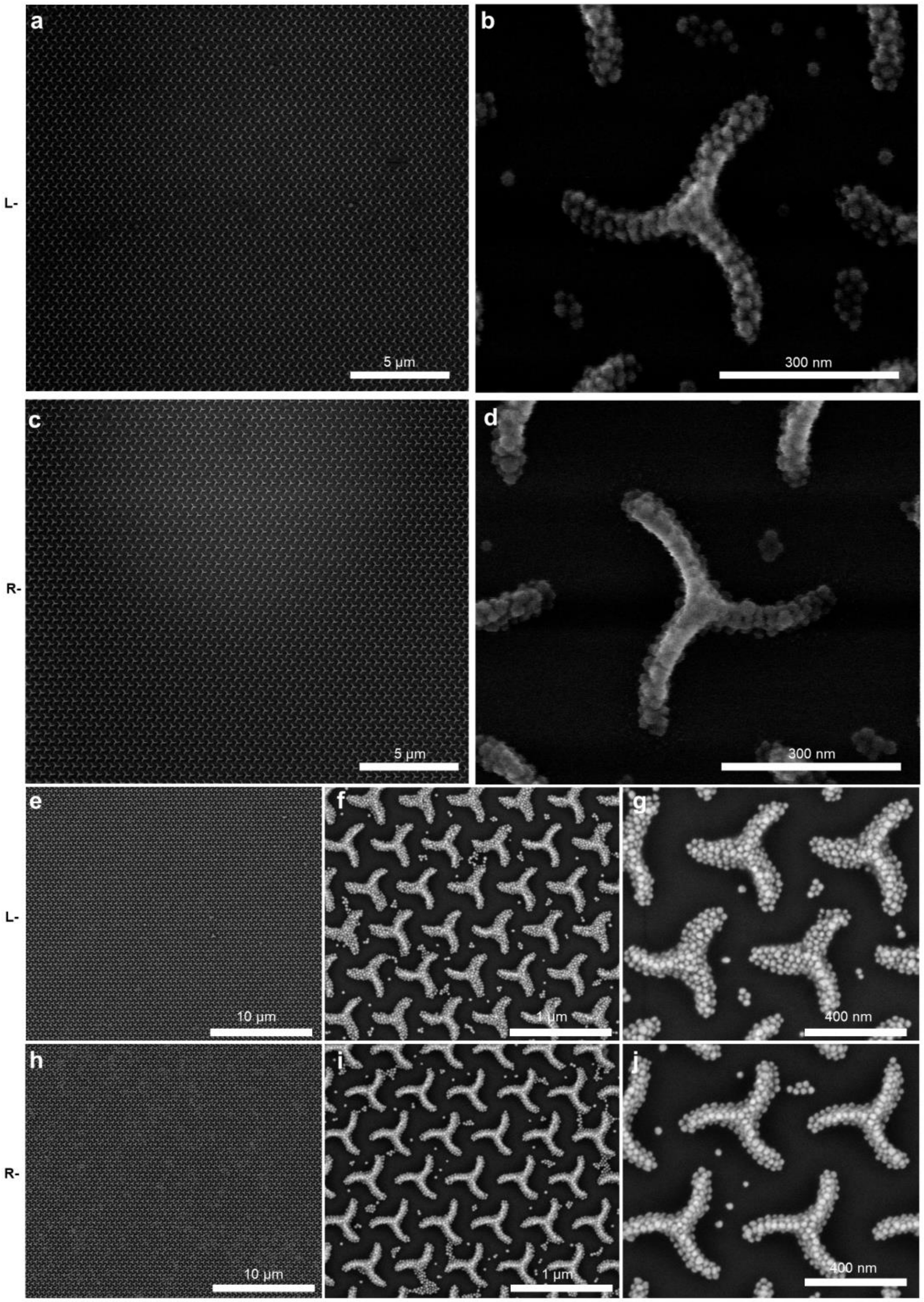

Supplementary Fig. 7. SEM images of 20 nm Ag nanospheres triskelion arrays. L- (**a**, **b**) and R- (**c**, **d**) triskelia arrays. SEM images of 27 nm Ag nanospheres L- (**e-g**) and R- (**h-j**) triskelia arrays.

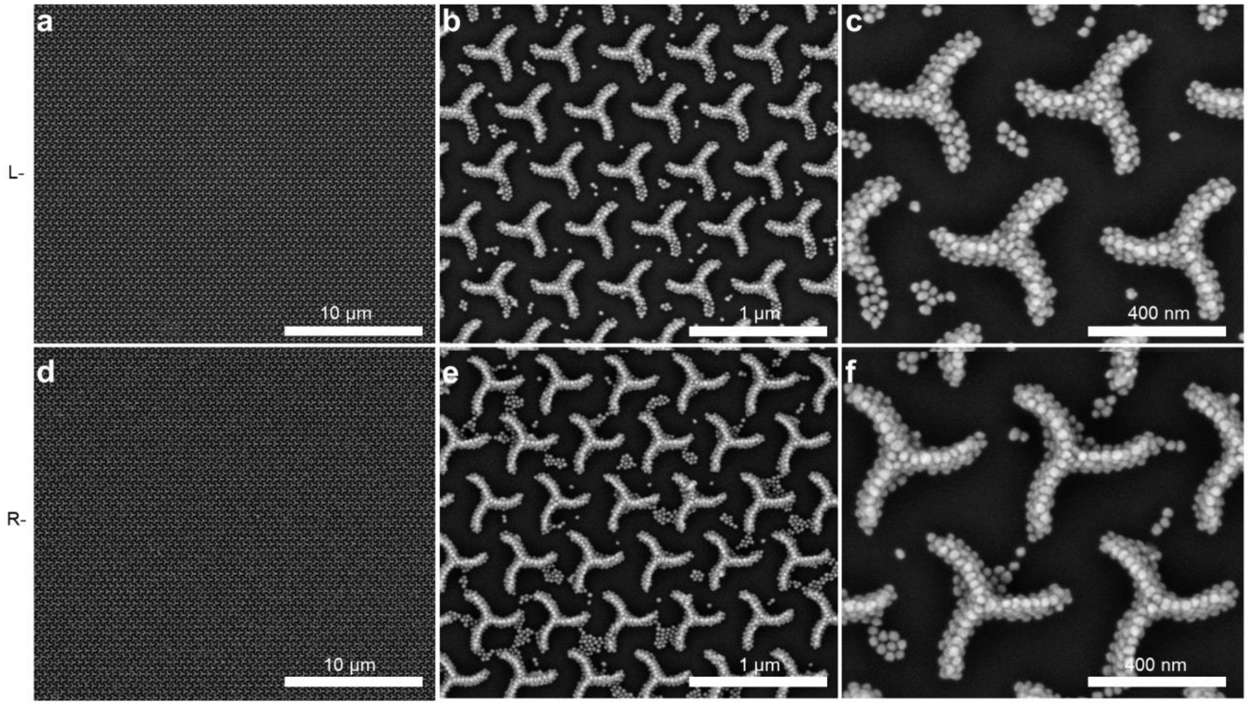

Supplementary Fig. 8. SEM images of 32 nm Ag nanospheres triskelion arrays. L- (**a**-**c**) and R- (**d**-**f**) triskelia arrays.

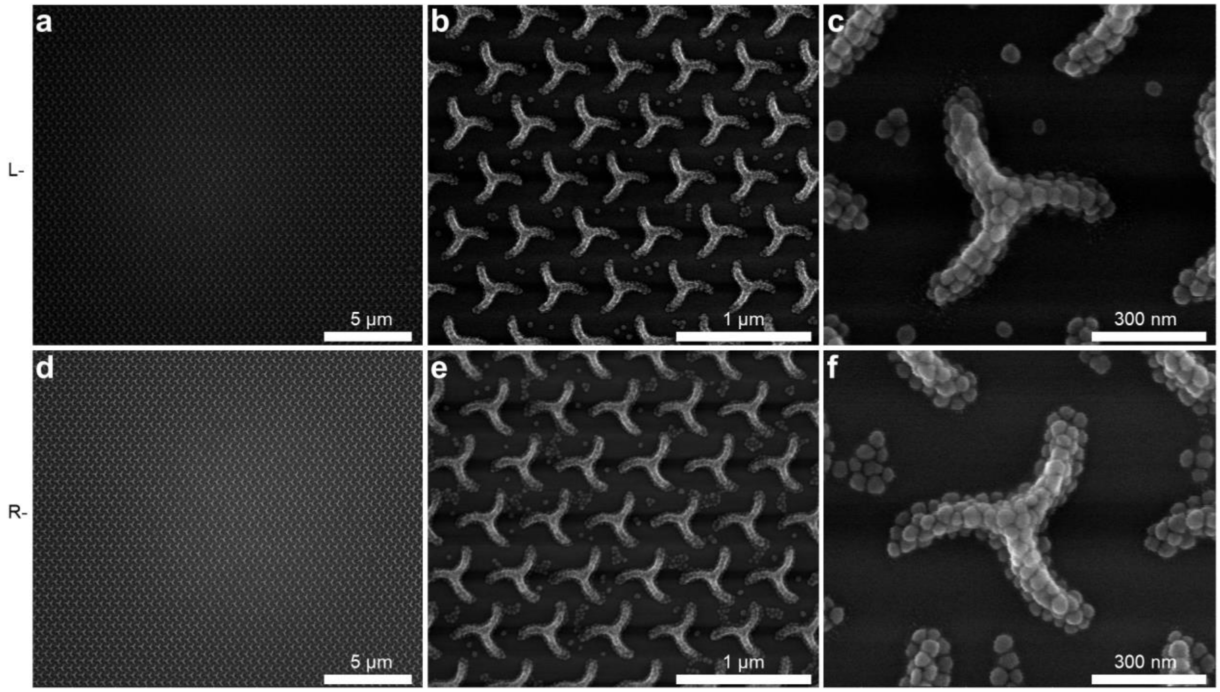

Supplementary Fig. 9. SEM images of 36 nm Ag nanospheres triskelion arrays. L- (**a**-**c**) and R- (**d**-**f**) triskelia arrays.

Nanostructures with gold colloids. Plasmonic films fabricated with gold colloids with different concentrations of [Au^0] (15, 30 and 50 mM) were tested to find the optimum optical response. As shown in Supplementary Figs. 10-12 for gold nanosphere sizes within 27-54 nm. For all the NPs sizes, at lower gold concentrations, the fine structure of the triskelion gradually became less resolved. For the 15 mM Au^0 case, gold nanospheres could not fill the whole triskelion pattern and one layer of colloids forms the triskelion pattern. It is important to note that even at Au^0 of 50 mM, there were no excess gold nanospheres diffused outside the triskelion patterns, and the entire arrays exhibited a notable self-assembly in each case.

As the size of the gold nanospheres used in the self-assembly increases (70 nm gold nanospheres), (Supplementary Fig. 13) the triskelion arms (75 nm wide in the PDMS molds) could not fit these particles. Therefore, most of the 70 nm gold nanospheres were located in the middle of the triskelion with almost no nanospheres observed out of the triskelia pattern. Unlike the single layer NPs triskelion pattern observed when low concentration made small gold nanospheres are used, for 70 nm gold colloid the nanospheres are located only at the center of each triskelion.

On the contrary, the structures composed of gold nanorods (Supplementary Fig. 14) could nicely fill the triskelion pattern and form well-organized triskelion arrays. The gold nanorods formed a head-to-tail self-assembly, with the short axis of the nanorods oriented along the small dimension.

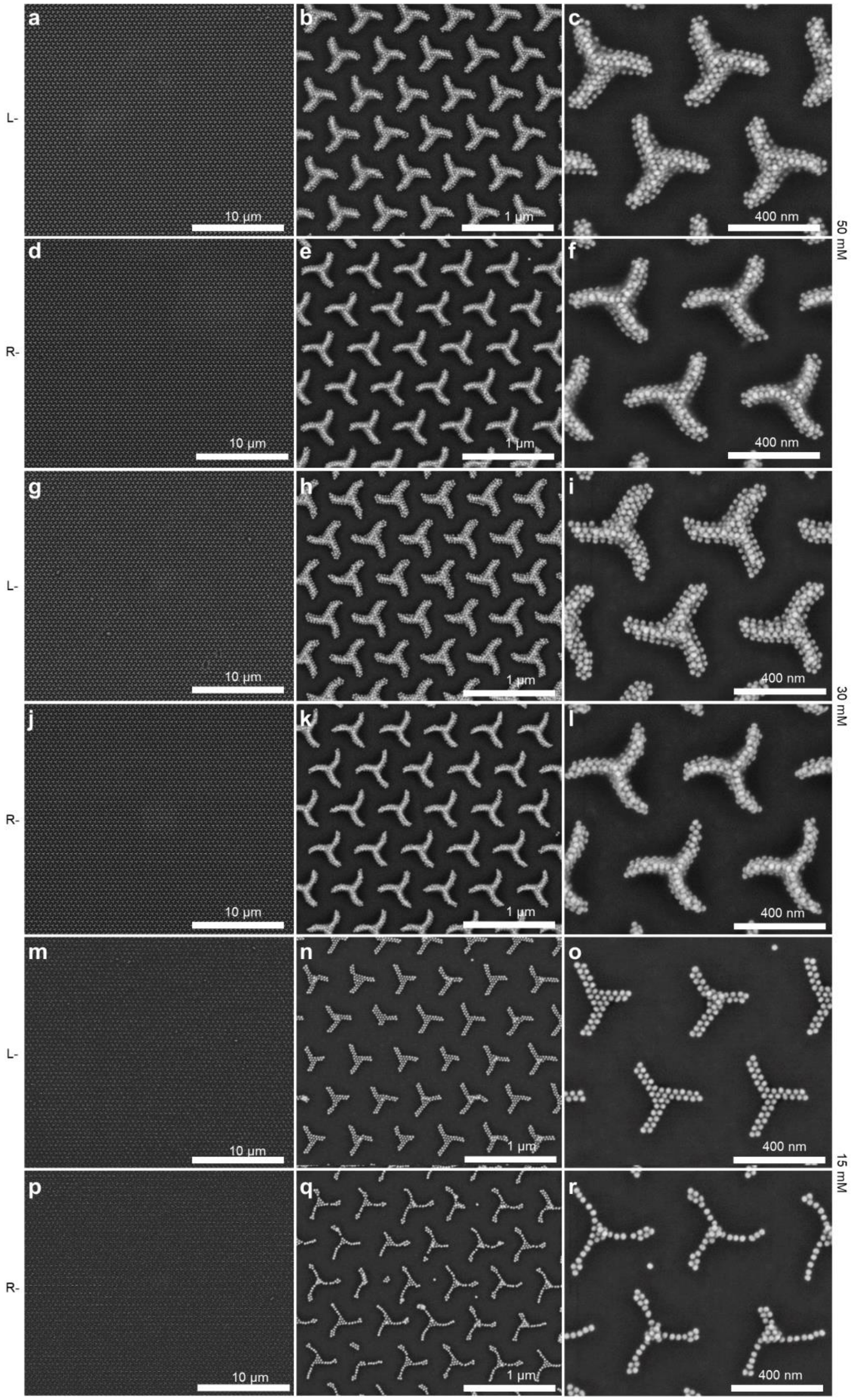

Supplementary Fig. 10. SEM images with triskelia pattern. Arrays made of 27 nm Au nanospheres with Au^0 concentration of 50 (**a**-**f**), 30 (**g**-**i**) and 15 (**m**-**r**) mM.

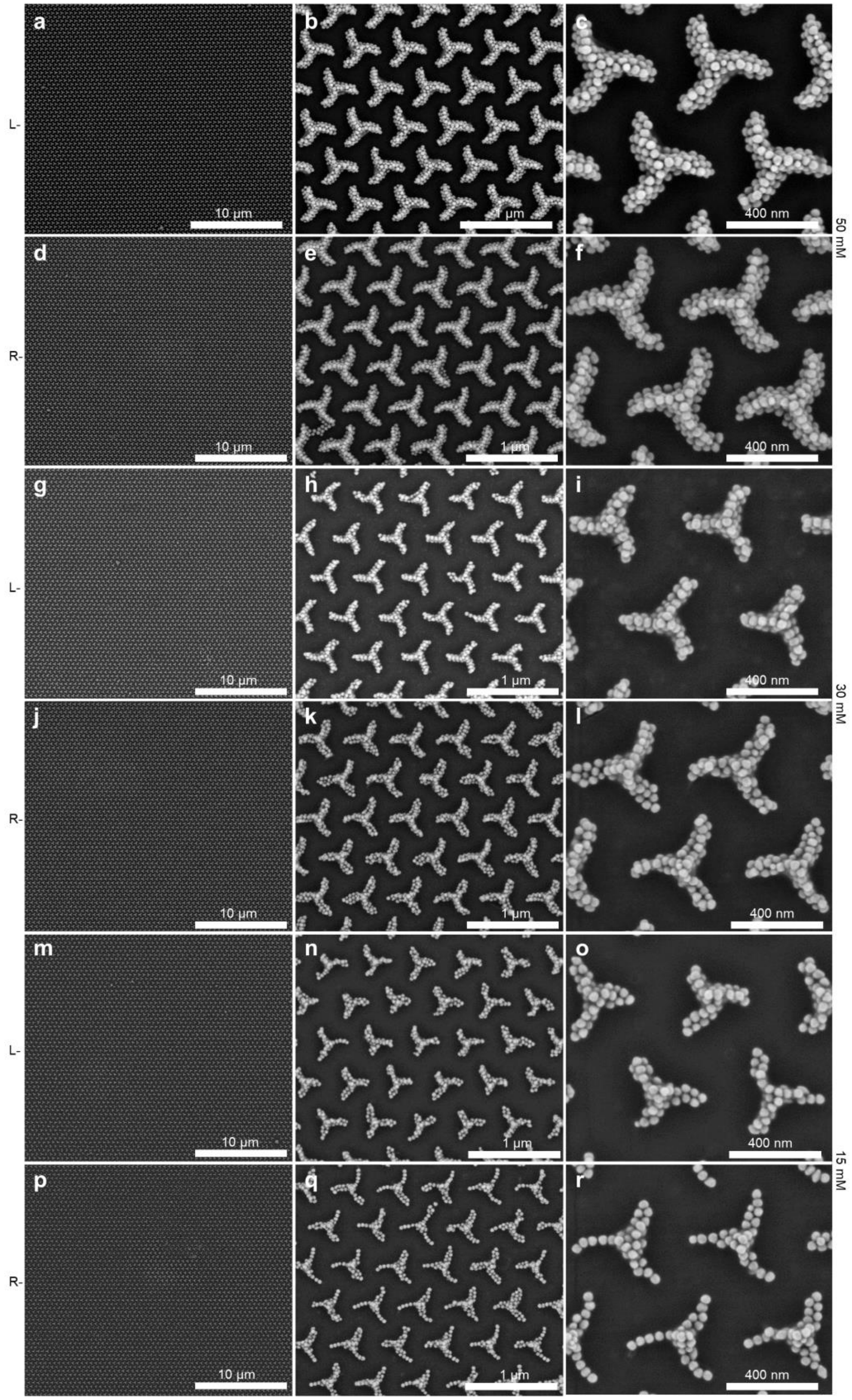

Supplementary Fig. 11. SEM images with triskelia pattern. Arrays made of 45 nm Au nanospheres with Au^0 concentration of 50 (**a**-**f**), 30 (**g**-**i**) and 15 (**m**-**r**) mM.

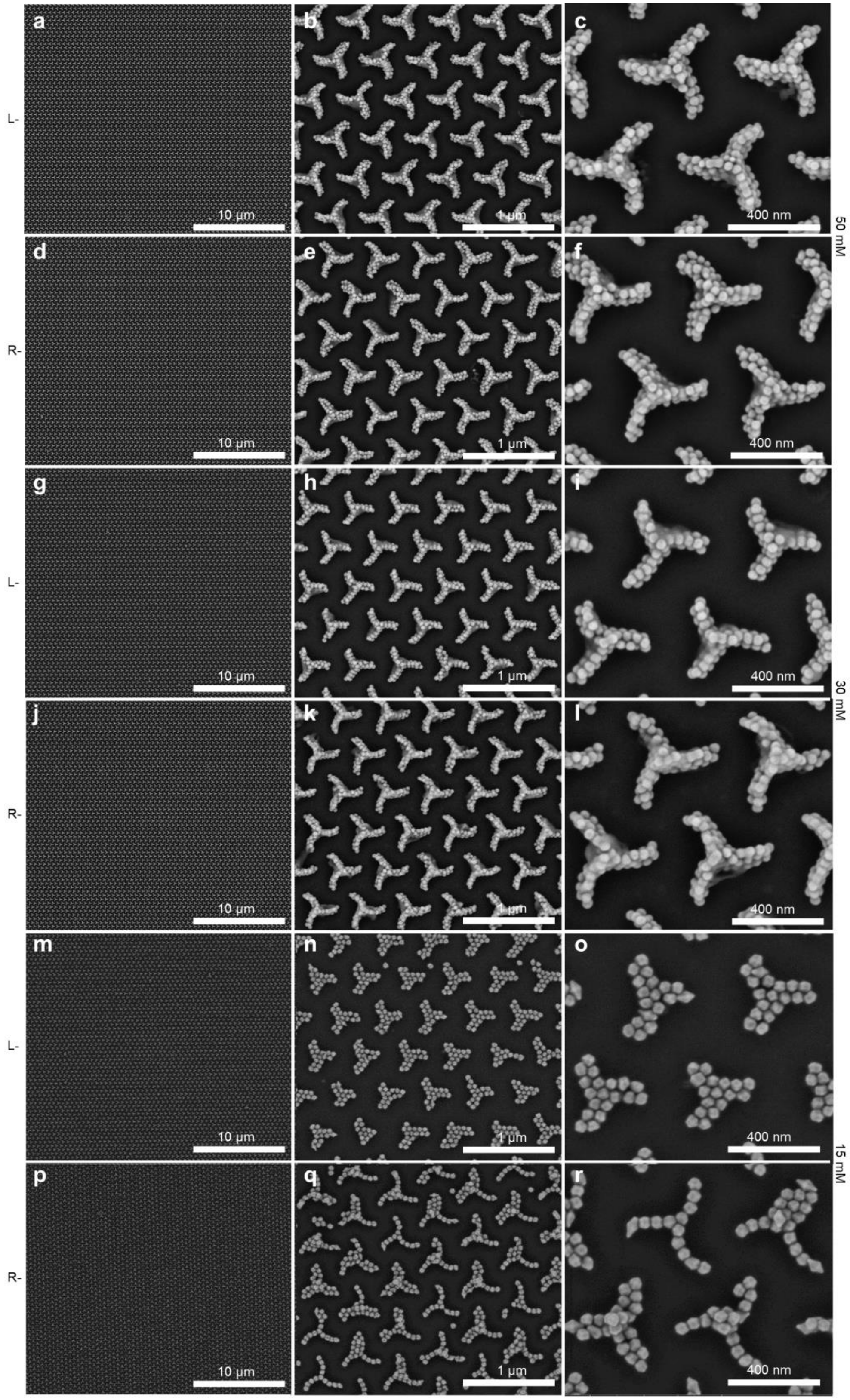

Supplementary Fig. 12. SEM images with triskelia pattern. Arrays made of 54 nm Au nanospheres with Au^0 concentration of 50 (**a**-**f**), 30 (**g**-**i**) and 15 (**m**-**r**) mM.

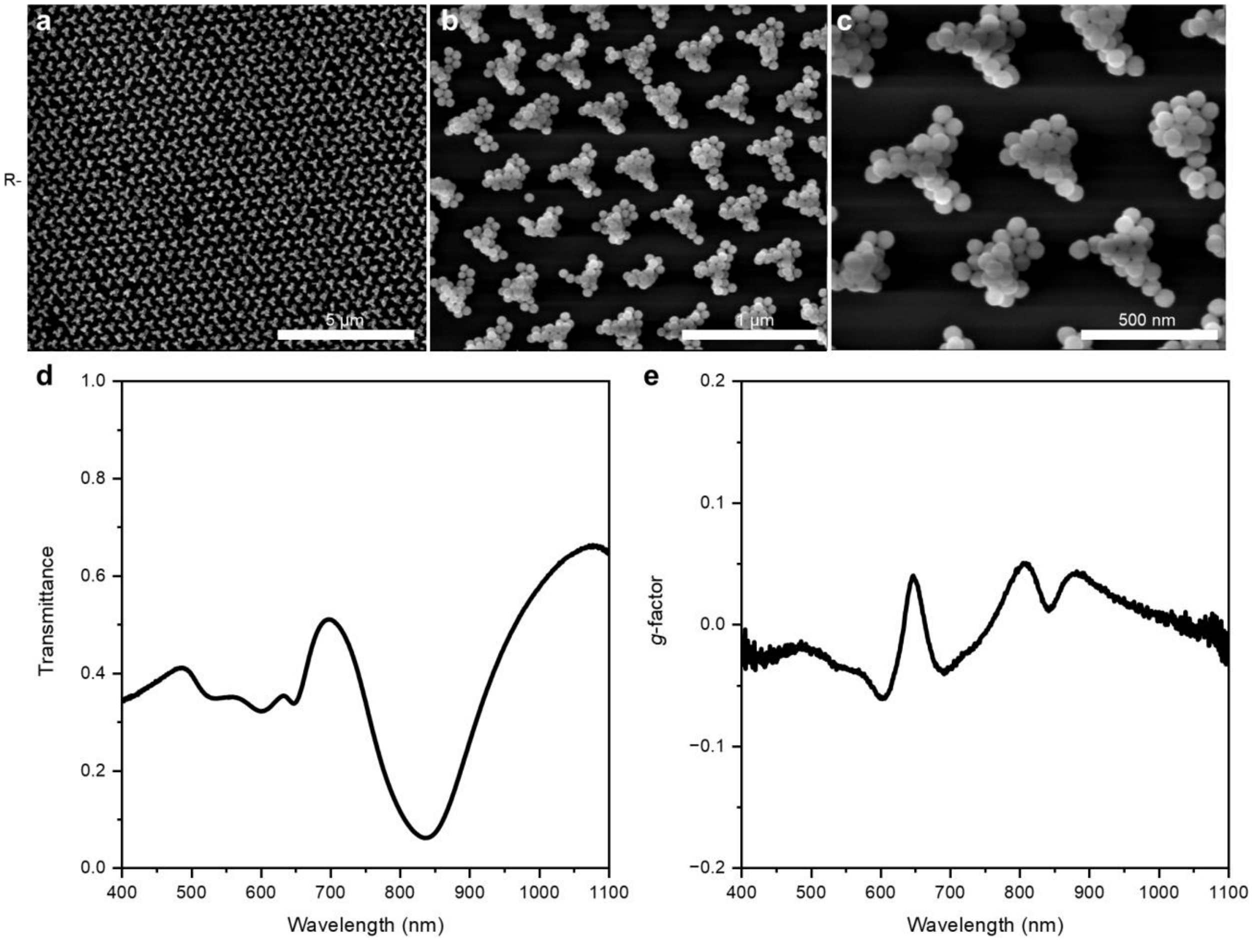

Supplementary Fig. 13. Triskelia pattern made of 70 nm Au nanospheres with 50 mM Au^0. (**a**-**c**) SEM images, (**d**) transmittance and (**e**) *g*-factor spectra. Source data are provided as a Source Data file.

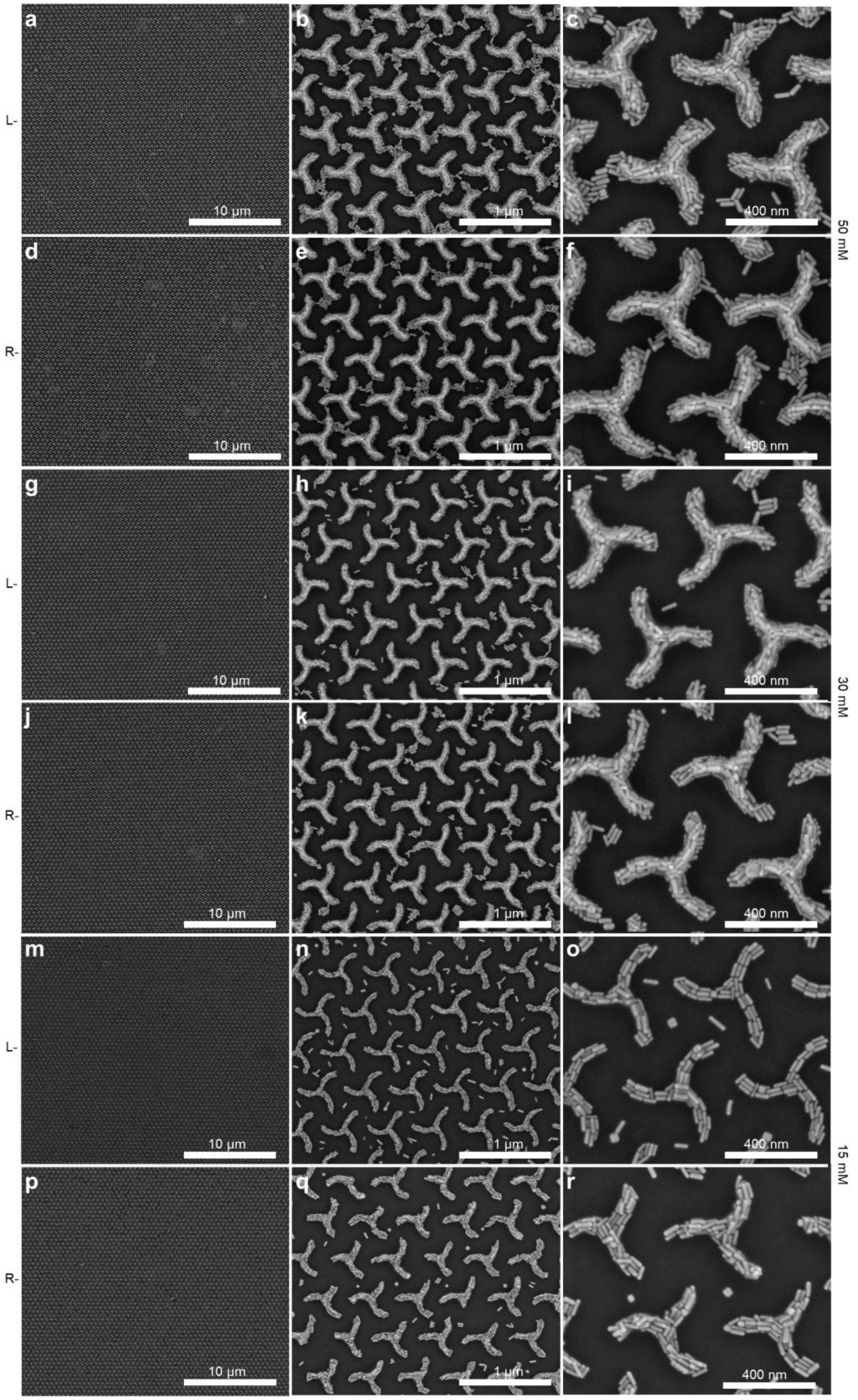

Supplementary Fig. 14. SEM images with triskelia pattern. Arrays made of Au nanorods with Au^0 concentration of 50 (**a**-**f**), 30 (**g**-**i**) and 15 (**m**-**r**) mM.

Suppl. Note 6. Comparison of different chirals geometries

Supplementary Fig. 15 presents a comparative analysis of the chiral properties of various architectures constructed from metallic colloids (30-50 nm gold nanospheres). In the SEM images, it can be observed that template-directed self-assembly facilitated the fabrication of highly ordered lattices with different chiral geometry unit cells. The geometries include G-shaped structures[6] (Supplementary Fig. 15a), tilted linear segment arrays (Supplementary Fig. 15b), gammadions (Supplementary Fig. 15c), and triskelions (Supplementary Fig. 15d). The G-shaped structures exhibit the largest lattice parameter (12 µm), the remaining three have lattice parameters of 500, 600 and 500 nm, respectively.

CD spectra (Supplementary Fig. 15e-h)in the Vis-NIR region demonstrate a pronounced dependence on the specific geometry. G-shaped structures exhibit broad CD bands with peak intensities around 40 mdeg. In contrast, segment line arrays display significantly higher maximum CD signals, exceeding 400 mdeg. Both gammadions and triskelions exhibit the highest CD responses with sharp, intense bands. The maximum CD signal for gammadions is centered near 900 nm in the NIR, whereas triskelions show the highest CD signal blue-shifted towards the visible region.

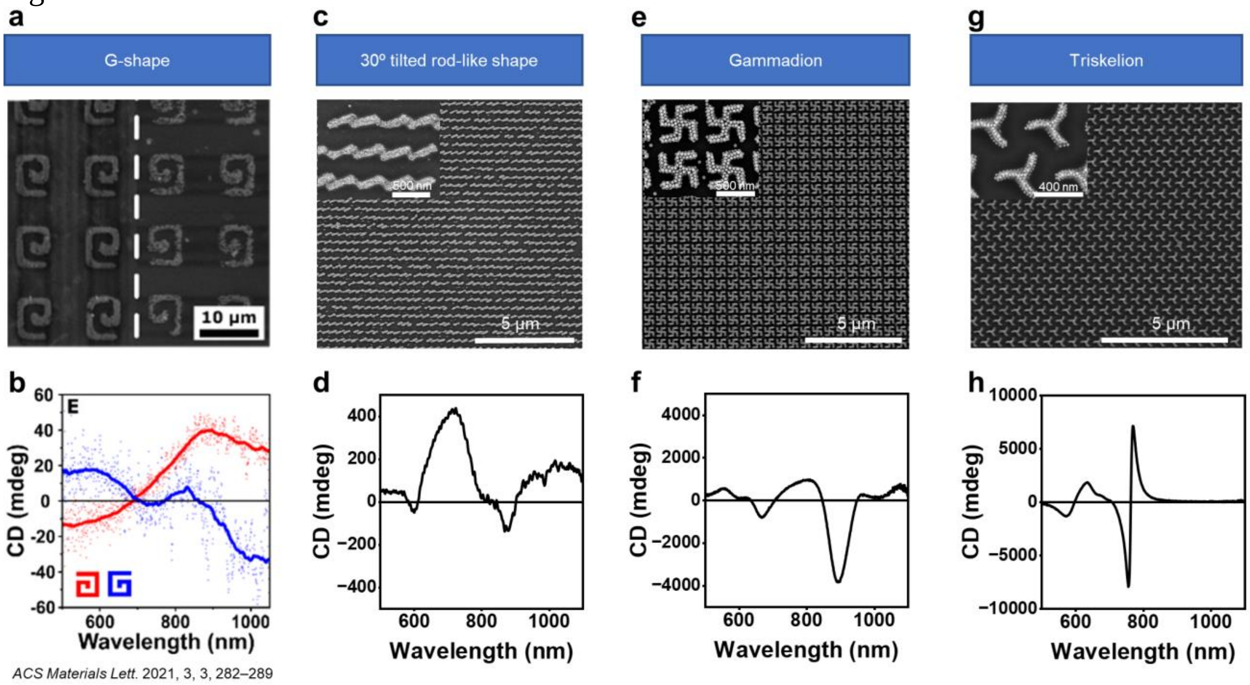

Supplementary Fig. 15. SEM images and CD spectra of various plasmonic architectures. Arrays constructed using spherical gold colloids. G-shape values are extracted from *N. Chiang* et al. Reproduced with permission from Large-scale soft-lithographic patterning of plasmonic nanoparticles. Chiang, N., Scarabelli, L., Vinnacombe-Willson, G. A., Pérez, L. A., Dore, C., Mihi, A., Jonas, S. J., Weiss, P. S. *ACS Mater. Lett.* **3**, 282-289 (2021). Copyright 2021 American Chemical Society. Source data are provided as a Source Data file.

We fabricated bilayer structures incorporating an epoxy SU8 interlayer between two stacked triskelion layers. As depicted in Supplementary Fig. 16a-c, the fabrication process involved: (Supplementary Fig. 16a) assembling nanoparticle arrays of L-triskelions on a glass substrate, (Supplementary Fig. 16b) coating the array with an SU8 layer using spin coating, and (Supplementary Fig. 16c) assembling a second layer of either L-triskelions or R-triskelions atop the SU8. Supplementary Figs. 16d, e present the transmittance spectra for each step. The initial array exhibited a prominent resonance at 750 nm. The coating with the SU8 layer significantly modified the optical response and a sharpening of the resonance due to its refractive index matching properties. Finally, adding the second nanoparticle layer resulted in a decrease in transmittance (since there is more metal). Overall, the chiral response is affected as shown in Supplementary Figs. 16f, g, however we did not observe a relevant increase in the *g*-factor in this case. Here we show the capability to create complex structures with our technique that can be explored in future works.

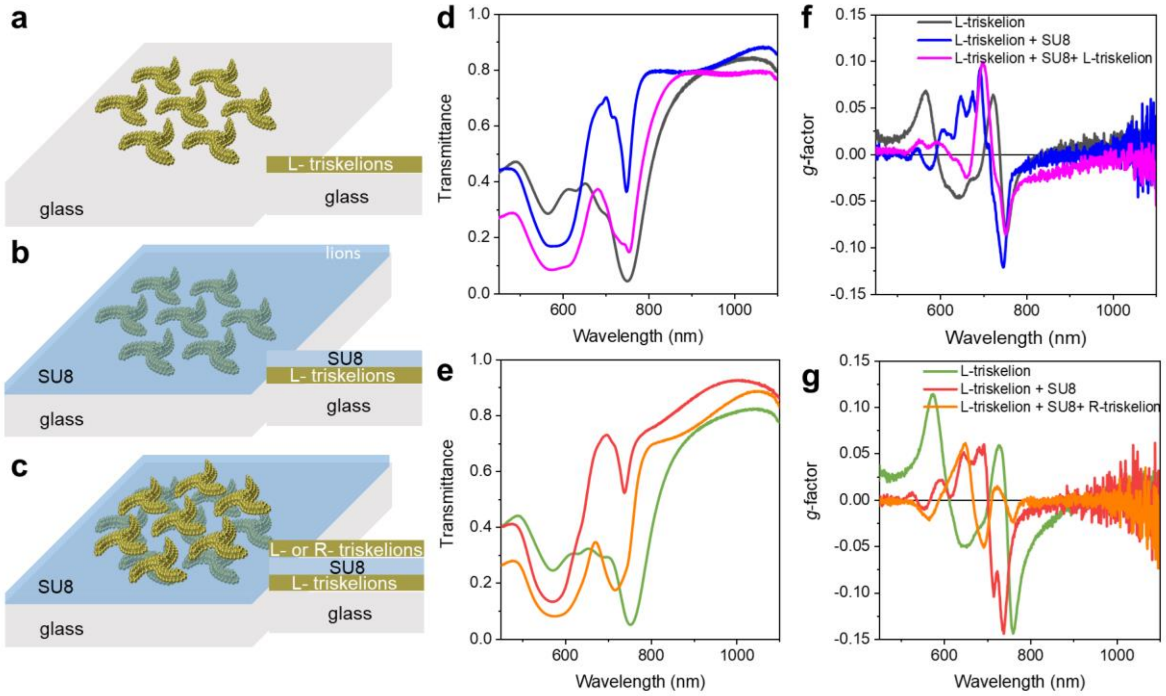

Supplementary Fig. 16. Schematics of the fabrication process and optical characterization of stacked plasmonic triskelions arrays. (**a**) The fabrication step shows: a) the first triskelion array, b) triskelion arrays covered with SU8 layer. c) the second triskelion arrays assembly. (**d**, **f**) Optical measurements from the sample (L-triskelions/SU8/L-triskelions) and (**e**, **g**) the sample (L-triskelions/SU8/R-triskelions). The triskelion arrays were made from colloids of Ag 27 nm. Source data are provided as a Source Data file.

Suppl. Note 7. Optical characterization set-up

CD set-up

CD measurements of the samples were obtained in a home-built optical setup schematically represented in Supplementary Fig. 17. Unpolarized light from a tungsten halogen lamp (Ocean Optics, HL-2000-HP, Florida, USA) was linearly polarized in the vertical direction using a calcite Glan-Thompson prism (GTH10M, Thorlabs). Superachromatic quarter waveplates (SAQWP05M-700 or SAQWP05M-1700, Thorlabs, depending on the spectral range) with the fast axis oriented at ±π/4 respect to the vertical direction generate left- and right- (L-/R-) circularly polarized (CP) light. The quarter waveplates were mounted onto rotation mounts (ELL14 Thorlabs). The light was focused on the sample using a 4× objective (NA = 0.1) (Supplementary Fig. 17a). The transmitted light was collected using a 4× objective and collimated to a fiber-coupled spectrophotometer (Ocean Insight, QEPro-FL (400-1100 nm) or NIR-Quest (1100-1800 nm). A similar setup was used for the angle-resolved characterization of the architectures (Supplementary Fig. 17b), the light was focused using a 10-cm working distance lens and the samples were placed on a rotational stage (Thorlabs, RP03/M, New Jersey, USA) to vary the illumination AOI in 1º steps. In transmittance characterization, air was used as reference.

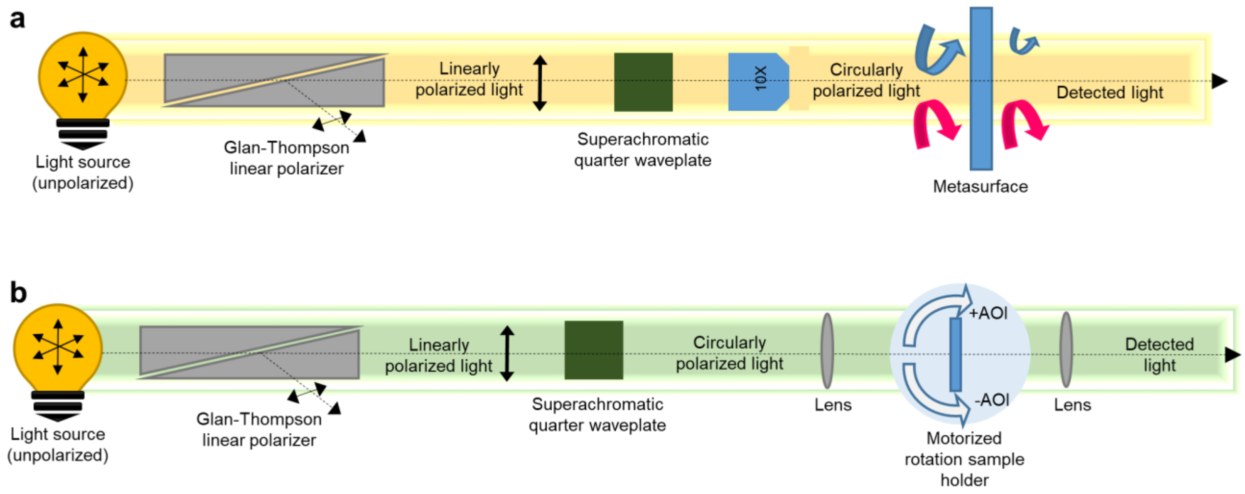

Supplementary Fig. 17. Experimental set-up used for characterizing the circular dichroism. With fixed (**a**) and rotating stage (**b**).

Unlike molecular systems, where circular dichroism is determined by the difference in the absorbance spectrum of LCP and RCP light (CD (ΔA) = A_{LCP} - A_{RCP} with A_{LCP} and A_{RCP}, representing the absorbance for LCP and RCP), in systems where light is not only transmitted ballistically but also is scattered or diffracted in a periodic array, the general extinction (E) (absorption + scattering) is considered. Therefore, the formula used in this context is given by:

$$\text{CD } (\Delta E) = E_{\text{LCP}} - E_{\text{RCP}} \tag{5}$$

The g-factor or Kuhn's dissymmetry factor is expressed as:

$$g \text{ factor} = \frac{\Delta E}{E} = 2\frac{(E_{\text{LCP}} - E_{\text{RCP}})}{E_{\text{LCP}} + E_{\text{RCP}}} = 2\frac{\left(-\log_{(T_{\text{LCP}})}\right) - \left(-\log_{(T_{\text{RCP}})}\right)}{\left(-\log_{(T_{\text{LCP}})}\right) + \left(-\log_{(T_{\text{RCP}})}\right)} \tag{6}$$

Here, E_{LCP} and E_{RCP} represent the extinction for left-circularly polarized and right-circularly polarized light, and T_{LCP} and T_{RCP} are the transmittance for LCP and RCP light, respectively.

CPL setup

As schematically shown in Supplementary Fig. 18, CPL was detected in a similar way by analyzing the polarization via an achromatic quarter waveplate (AQWP10M-580) at ±π/4 that converted each circular polarization into two orthogonal linear polarization states. Both polarizations were then directed to a Glan-Thompson linear polarizer, which allowed vertical polarization to pass through while horizontal polarization was reflected at 90°, thus selecting a single-handed polarization state along the setup optical axis. Either polarization chirality could be selected by placing the quarter-wave plate at a position of ±45° relative to the vertical axis of the linear polarizer. The PL was excited using a pulsed supercontinuum laser (Fianium SC400) emitting at 775 nm. The laser light source was cleaned using a short pass filter at 800 nm (FESH0800, Thorlabs) to avoid any parasitic light from the excitation source overlapping with the collected PL of the emitting dye. The sample is placed in a rotating stage (RP03, Thorlabs) at the focal plane of a pair of 10× objectives (Olympus RMS10X, Thorlabs) of 0.25NA to enhance the collected signal. However, the PL is filtered for the ballistic direction using an iris (SM1D25, Thorlabs) to account only for the dissymmetric PL emission at the given direction. The laser excitation source is then filtered with a longpass filter at 800 nm (FELH0800, Thorlabs) to detect only the signal coming from the PL. In this case, the asymmetric emission of each polarization chirality would be characterized by the photoluminescence asymmetry factor (g_{lum}) defined as in previous studies according to:

$$g_{\mathrm{lum}} = \frac{\Delta I}{I} = 2\frac{(I_{\mathrm{LCP}} - I_{\mathrm{RCP}})}{I_{\mathrm{LCP}} + I_{\mathrm{RCP}}} \quad (7)$$

Where I_{LCP} and I_{RCP} are the emitted PL intensities for left and right circularly polarized luminescence and I is the total PL intensity.

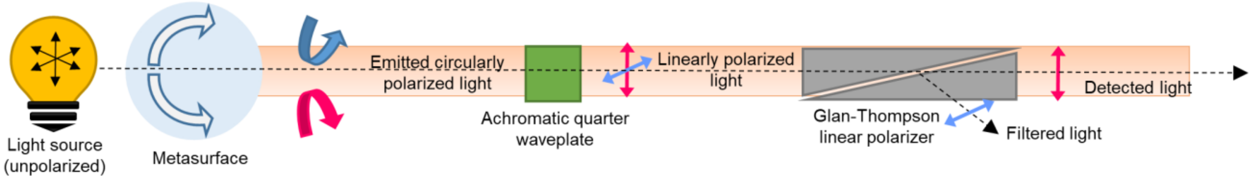

Supplementary Fig. 18. Experimental set-ups used for collecting the circularly polarized light.

Suppl. Note 8. Supplementary CD spectra

Evolution of the optical properties with varying concentration of metal nanospheres

The transmittance and *g*-factor spectra of samples with different concentrations were explored. As shown in Supplementary Figs. 19b, d, within the range of 15-50 mM of Au^0, the transmittance at the SLRs and LSPRs gradually decreased and redshifted (more pronounced for 45 nm NPs) and the *g*-factor gradually increased by increasing colloid concentration. Beyond this range, further increasing the concentration rendered no remarkable changes in the transmittance and *g*-factor. This is because, when the concentration of Au^0 was 50 mM, the triskelion pattern was basically saturated, and there was no extra place to correctly fit more nanocolloids (as can be seen from

SEM images with Supplementary Figs. 11-13), so with the increase of the concentration of Au^0, the excess nanocolloids would spread outside the triskelion pattern, affecting the overall shape of the pattern and *g*-factor.

The trend in the case of silver nanoparticles is similar with a decreased transmittance with the increasing concentration (Supplementary Figs. 19e, f). Interestingly, with the increase of concentration of Ag^0, the *g*-factor of triskelion arrays showed an increase, and this was consistent with the results for gold nanoparticles.

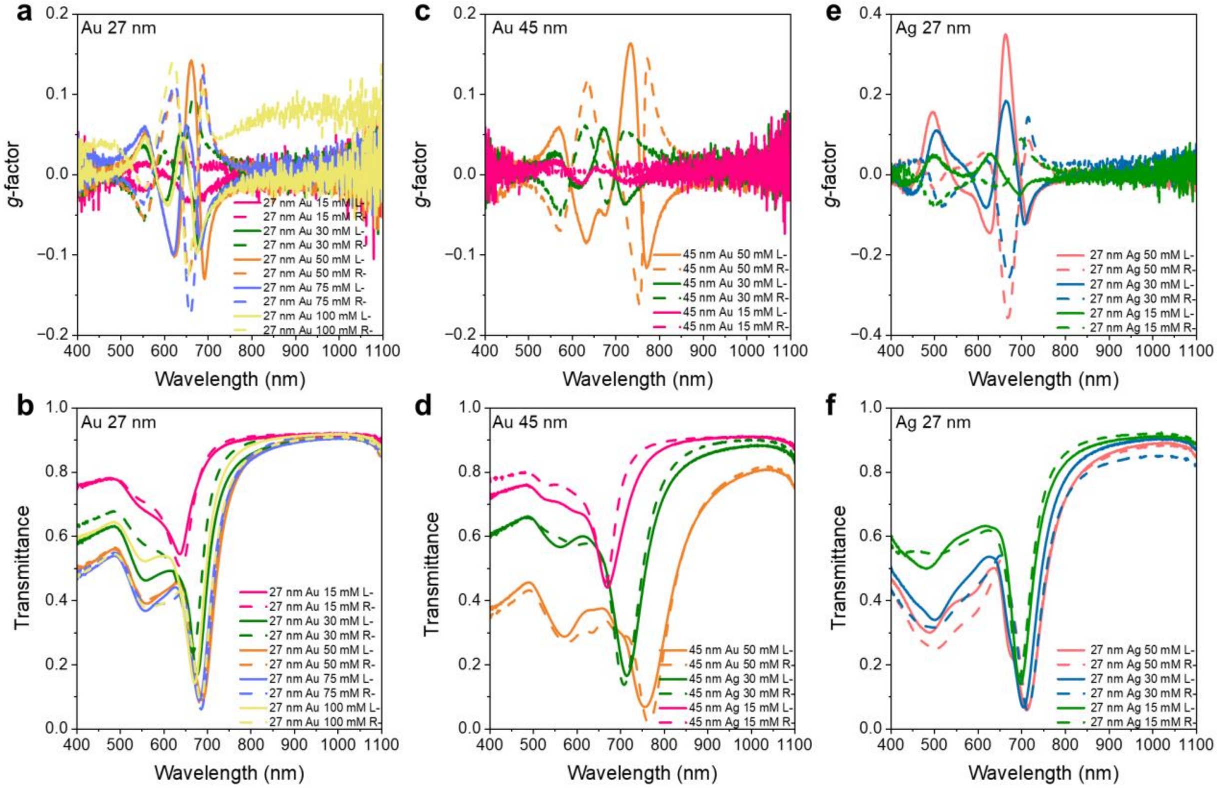

Supplementary Fig. 19. **Transmittance and circular dichroism characterization.** Circular and (**a**, **c** and **e**) *g*-factor and (**b**, **d** and **f**) transmittance for triskelion arrays with different concentration of gold and silver nanospheres. Source data are provided as a Source Data file.

LCP and RCP transmittance spectra

The related LCP, and RCP transmittance measurements used in Fig. 3 for different colloids at the optimum concentration are shown in Supplementary Figs. 20-22.

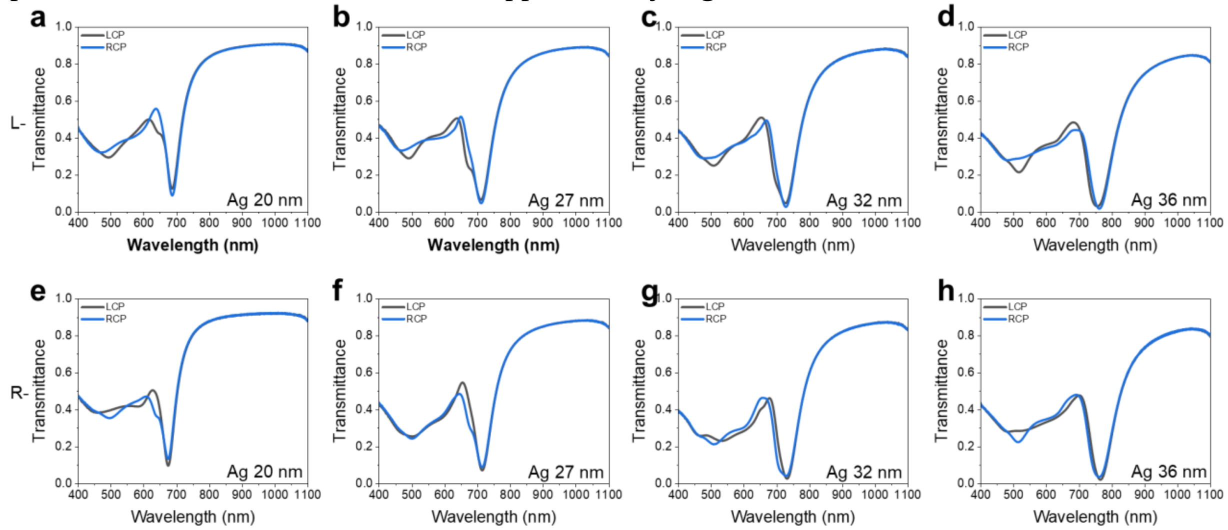

Supplementary Fig. 20. Transmittance spectra (LCP (black lines) and RCP (blue lines)) for L- (**a-d**) and R- (**e-h**) triskelion arrays with different size of silver nanospheres with Ag^0 = 50 mM. Source data are provided as a Source Data file.

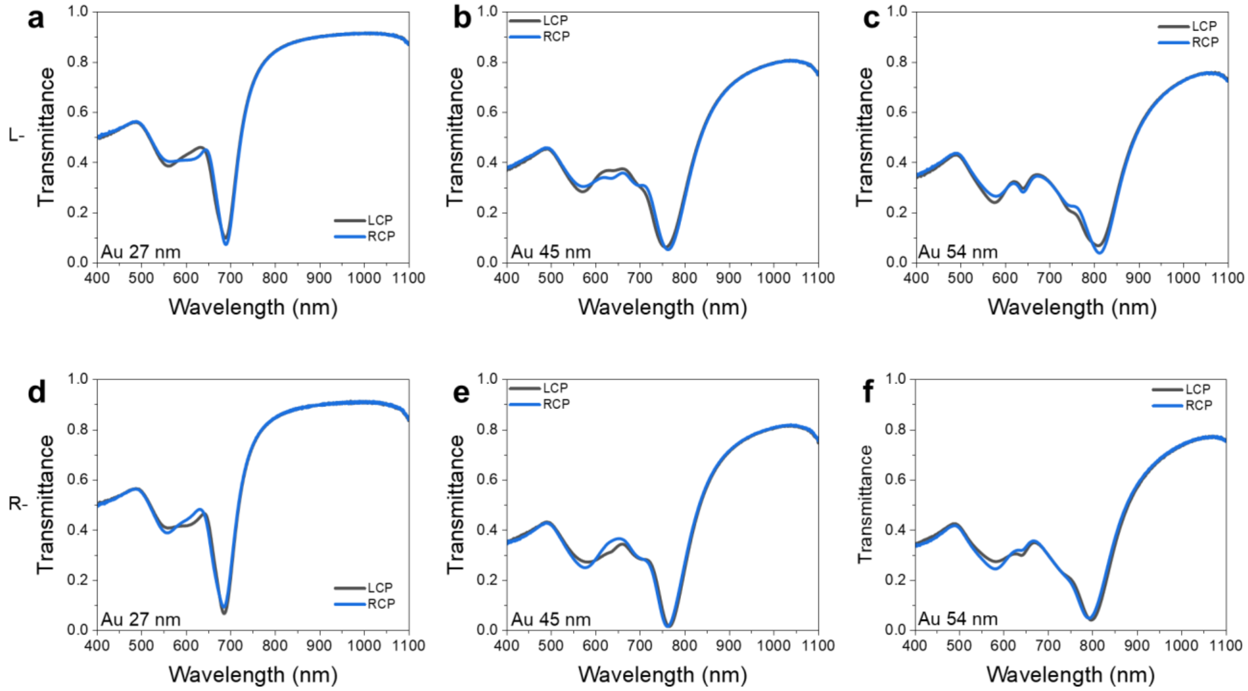

Supplementary Fig. 21. Triskelion arrays LCP and RCP transmittance spectra. Transmittance spectra (LCP (black lines) and RCP (blue lines)) for L- (**a-c**) and R- (**d-f**) triskelion arrays with different size of gold nanospheres with Au^0 = 50 mM. Source data are provided as a Source Data file.

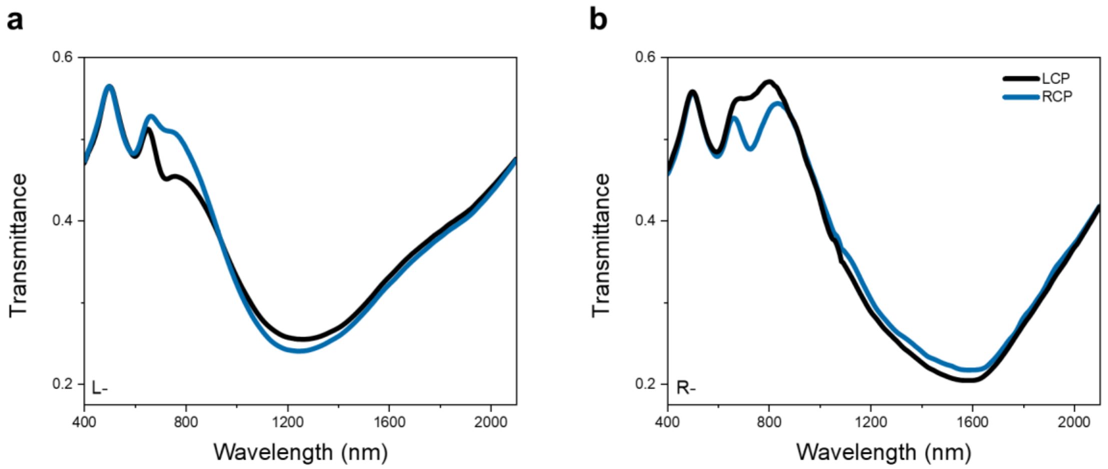

Supplementary Fig. 22. Nanorods triskelions arrays optical characterization. Transmittance spectra (LCP and RCP) for (**a**) L- and (**b**) R- triskelion arrays (gold nanorods) with Au^0 50 mM. Source data are provided as a Source Data file.

Evolution on the optical response of the arrays onto substrates with different refractive index

27 nm Ag nanospheres were used to check the optical performance on substrates with different refractive index (Supplementary Fig. 23). Increasing the refractive index of the substrate produced a redshift in the SLR of the triskelion arrays (Supplementary Fig. 23a). Similarly, the *g*-factors redshifted accordingly but also showed a higher *g*-factor value when the refractive index contrast between the substrate and the superstrate (air) is higher. This further supports the idea that the substrate induces an asymmetry that enhances the dichroism of the structure.

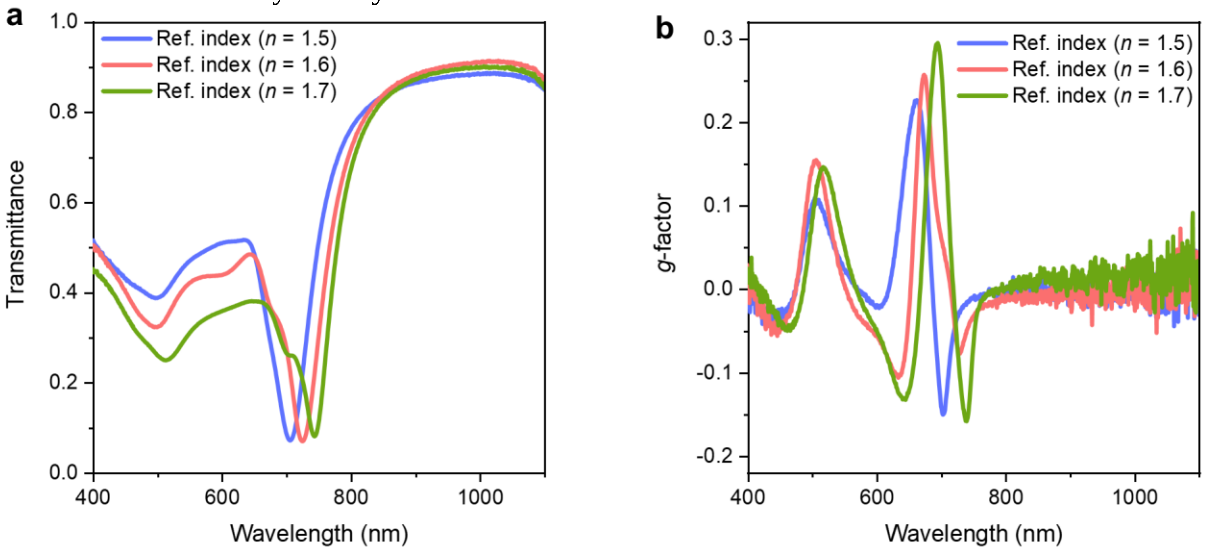

Supplementary Fig. 23. Substrate refractive index influence on the optical properties. (**a**) Transmittance and (**b**) *g*-factor spectra of triskelion arrays of 27 nm Ag NPs on different refractive index substrates. Source data are provided as a Source Data file.

Sample azimuthal angle effects – Linear Dichroism contribution

The transmittance and CD spectra of a triskelia pattern composed of 45 nm gold nanospheres was characterized at multiple azimuthal angles (these measurements were all at normal incidence), the results are shown in Supplementary Fig. 24. It can be observed that both transmittance, and *g*-

factor were not affected by rotating the sample from 0° to 315°, proving that the linear polarization effects of the triskelion CD response is negligible.

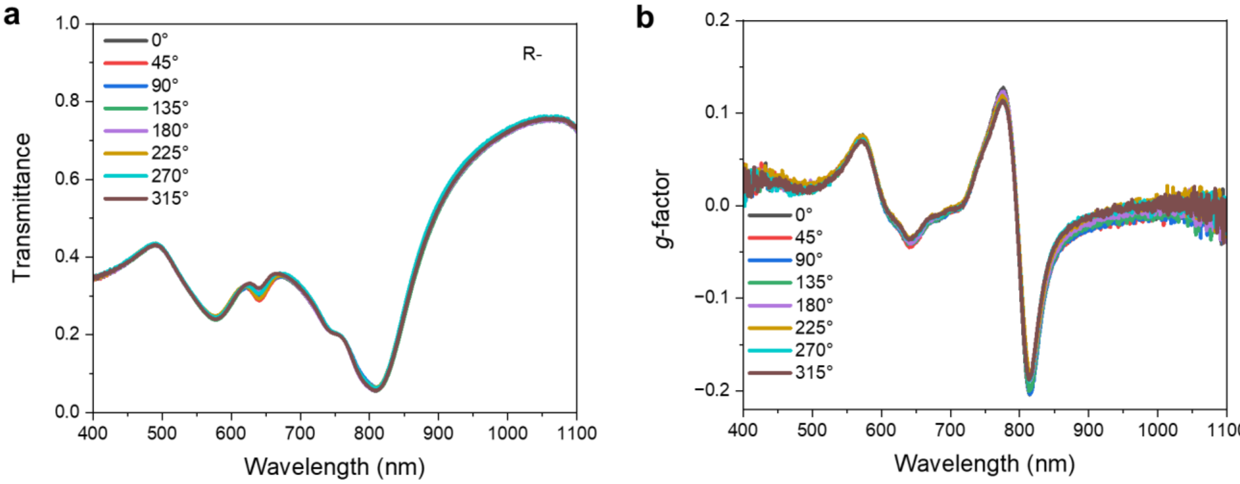

Supplementary Fig. 24. Azimuthal angles characterization. (**a**) Transmittance using linearly polarized light and (**b**) *g*-factor spectra of an R-triskelion pattern of 54 nm gold nanospheres at different azimuthal angles. Source data are provided as a Source Data file.

Validation of 3D behavior structures: Reversing direction of incident light

To further characterize the structure of triskelion arrays composed of colloids, we conducted transmittance and CD measurements of the triskelion array composed of 32 nm silver nanospheres for light impinging from both sides of the sample: light going through the substrate (glass) first or light impinging from air / array. The result is shown in Supplementary Fig. 25, the same peak shape and value could be obtained from the detection in two different directions. This proved that the triskelion array composed of colloids behaves like a 3D structure in terms of chirality.

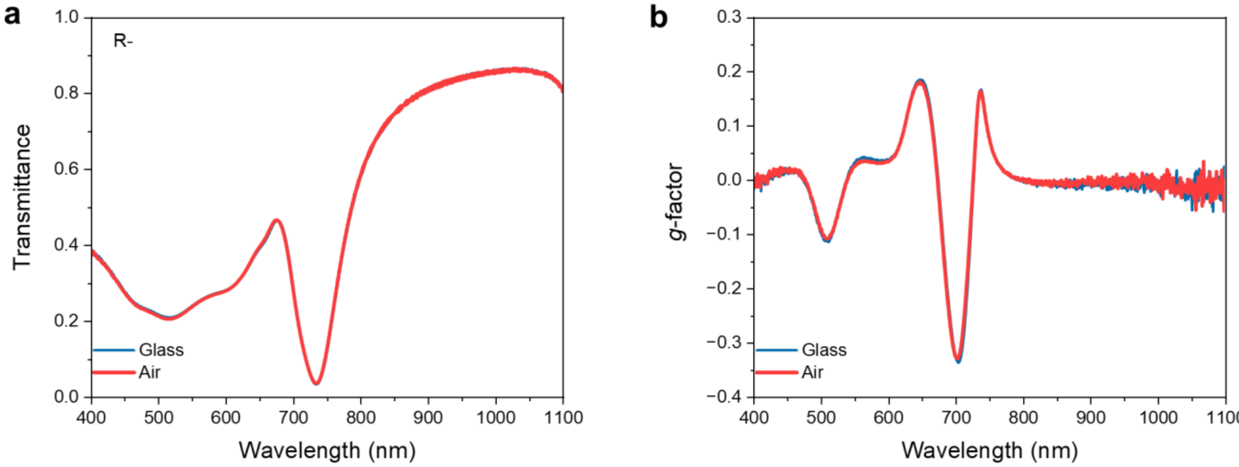

Supplementary Fig. 25. Light incidence direction. (**a**) Transmittance and (**b**) *g*-factor spectra of 32 nm Ag nanospheres obtained from different light direction (light impinging from the substrate side (glass) or from the air side. Source data are provided as a Source Data file.

Optical characteristics of the lattices with index-matching conditions

After adding index matching oil (n = 1.518) on the gold and silver triskelia arrays, the structures exhibited quite different optical characteristics regarding CD. As can be seen from Supplementary Figs. 26a, b, for Ag nanospheres, after IM, the g-factor at normal incidence decreased significantly regardless of the colloid size. In the case of gold, arrays made with small Au nanospheres (Supplementary Figs. 26c, d) after IM followed the same trend as for Ag. However, the g-factor for 54 nm Au nanospheres increased significantly.

The FWHM of the transmittance spectra, decreased significantly after IM, no matter what kind of colloids were used. In addition, the LSPR of Ag nanospheres and 27 nm Au nanospheres showed a redshift. Conversely, the 54 nm Au nanospheres produced a small blue shift.

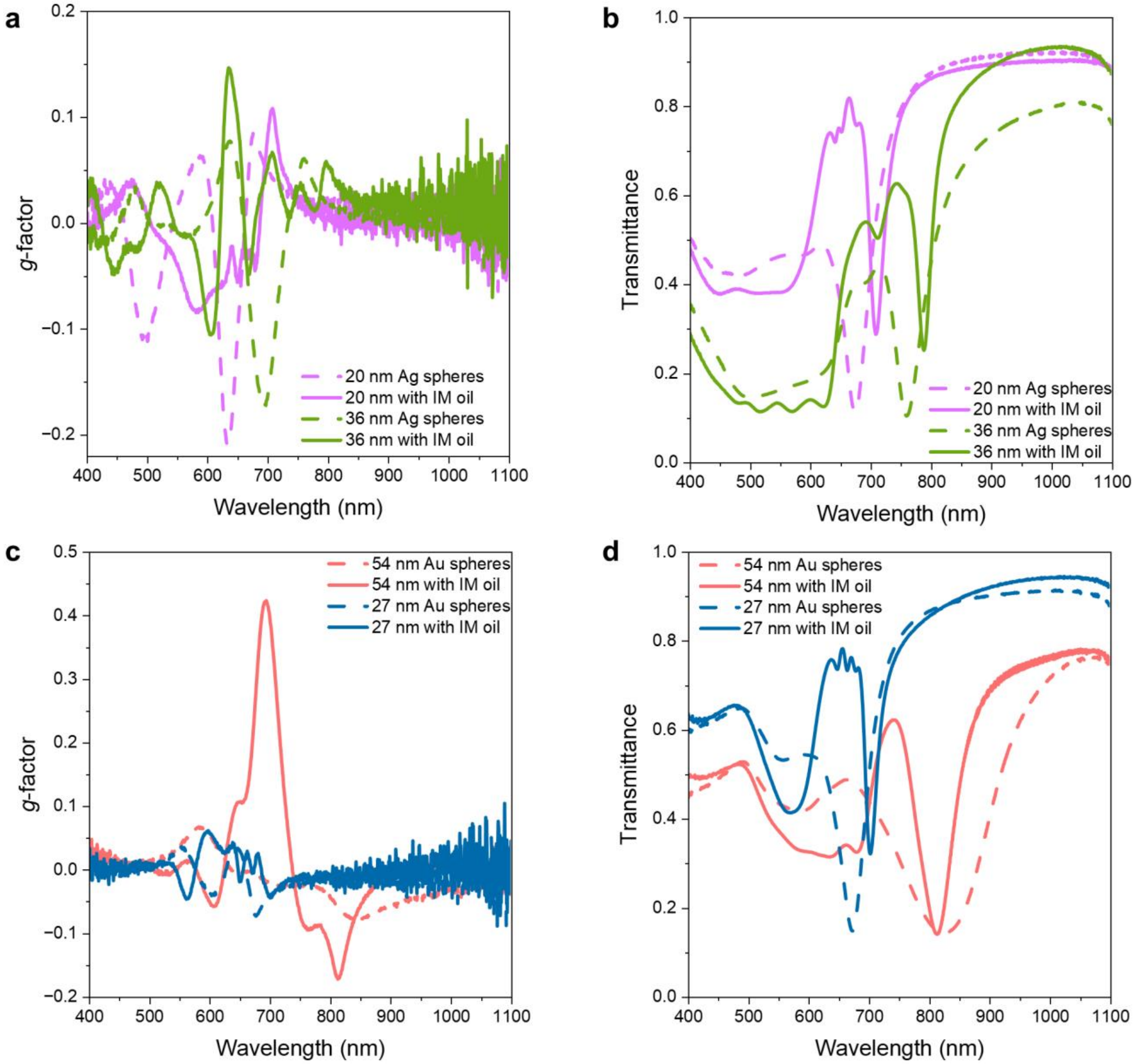

Supplementary Fig. 26. Index matched samples. g-factor and transmittance spectra for triskelion arrays made of (**a**, **b**) Ag and (**c**, **d**) Au nanospheres before and after IM oil (n = 1.518). Source data are provided as a Source Data file.

Suppl. Note 9. Angle-resolved characterization --- CD response of a hexagonal lattice of triskelia vs. disks

Angular resolved transmittance characterization for RCP, LCP and s-polarized incident light in the Γ-M and Γ-K direction of the hexagonal lattice was carried out employing the custom optical setups outlined in Suppl. Note 6. The transmitted light intensity was examined as a function of wavelength, resolved at 1º increments of the AOI, spanning from -45º to +45º. Subsequently, maps were generated for each incident light polarization, along with maps depicting the chiral properties represented by the *g*-factor. These maps are displayed with the component of *k* parallel to the sample surface on the x-axis and energy on the y-axis.

The chiral response presented in Supplementary Fig. 27 for Au triskelion arrays is entirely dependent on the differential coupling to the plasmonic surface lattice mode by circularly polarized light, depending on its handedness. However, the mere presence of a hexagonal diffraction grating composed of NP clusters is not sufficient for the observation of high chiral response. As shown in Supplementary Fig. 28, when arrays composed of NPs "cylindrical" clusters arranged in a hexagonal lattice, the response with respect to polarization state cannot be differentiated, with *g*-factor values having maxima of barely ±0.04. Even when the observed SLRs are intense and sharp, the difference of the LCP and RCP trend is negligible compared with triskelia arrays, indicating that the interaction between the chiral modes generated in the unit cells and the lattice is responsible for the giant response of the system.

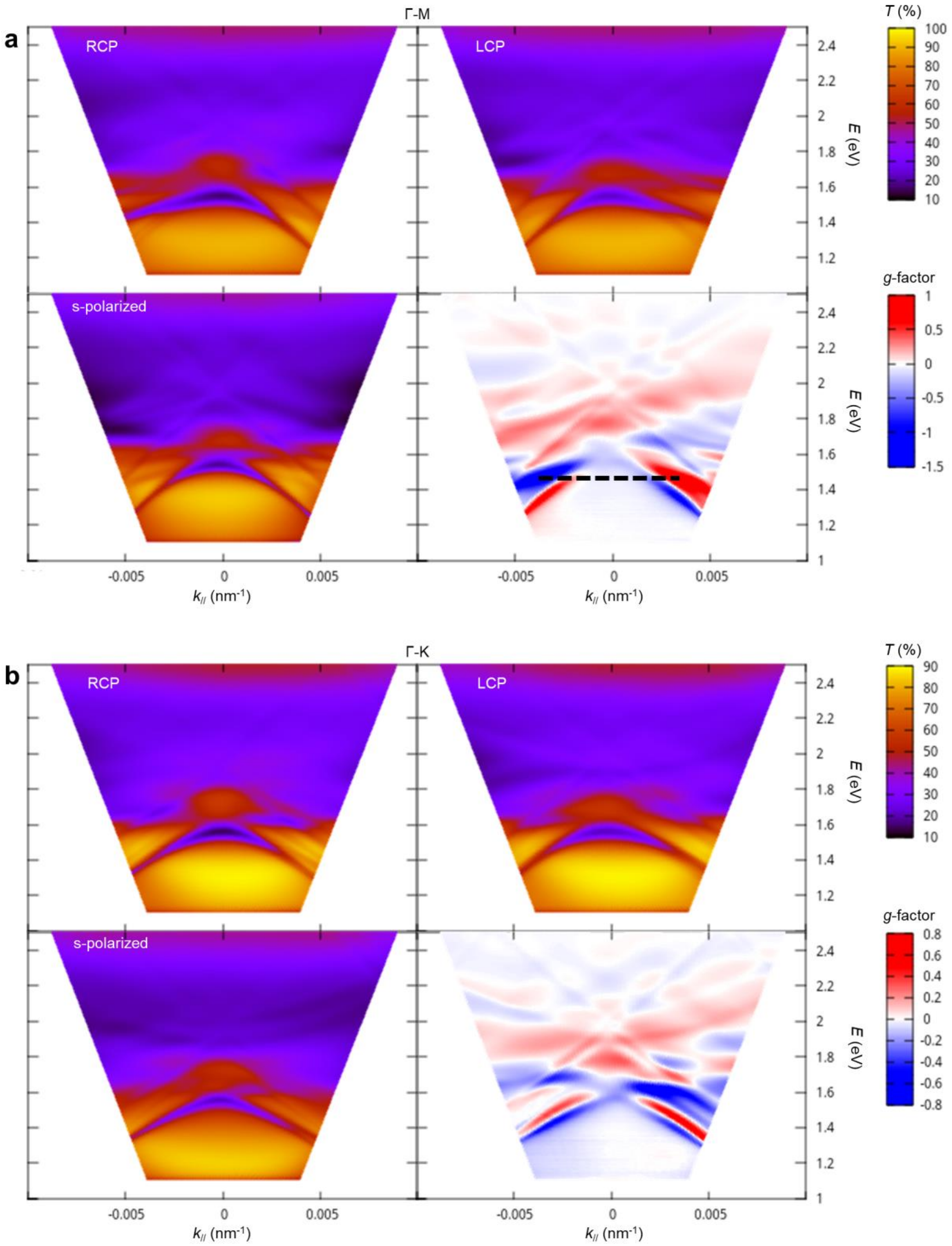

Supplementary Fig. 27. Angle-resolved optical response. 45 nm gold triskelia hexagonal arrays with SU8 in the (**a**) Γ-M and (**b**) Γ-K directions. Angular distribution maps in Energy versus wavevector for transmittance under RCP, LCP, s-polarized light and *g*-factor maps are shown. The dash black line at 1.46 eV in the *g*-factor map (**a**) represent the IR-140 emission energy, as shown in (Fig. 5f-h).

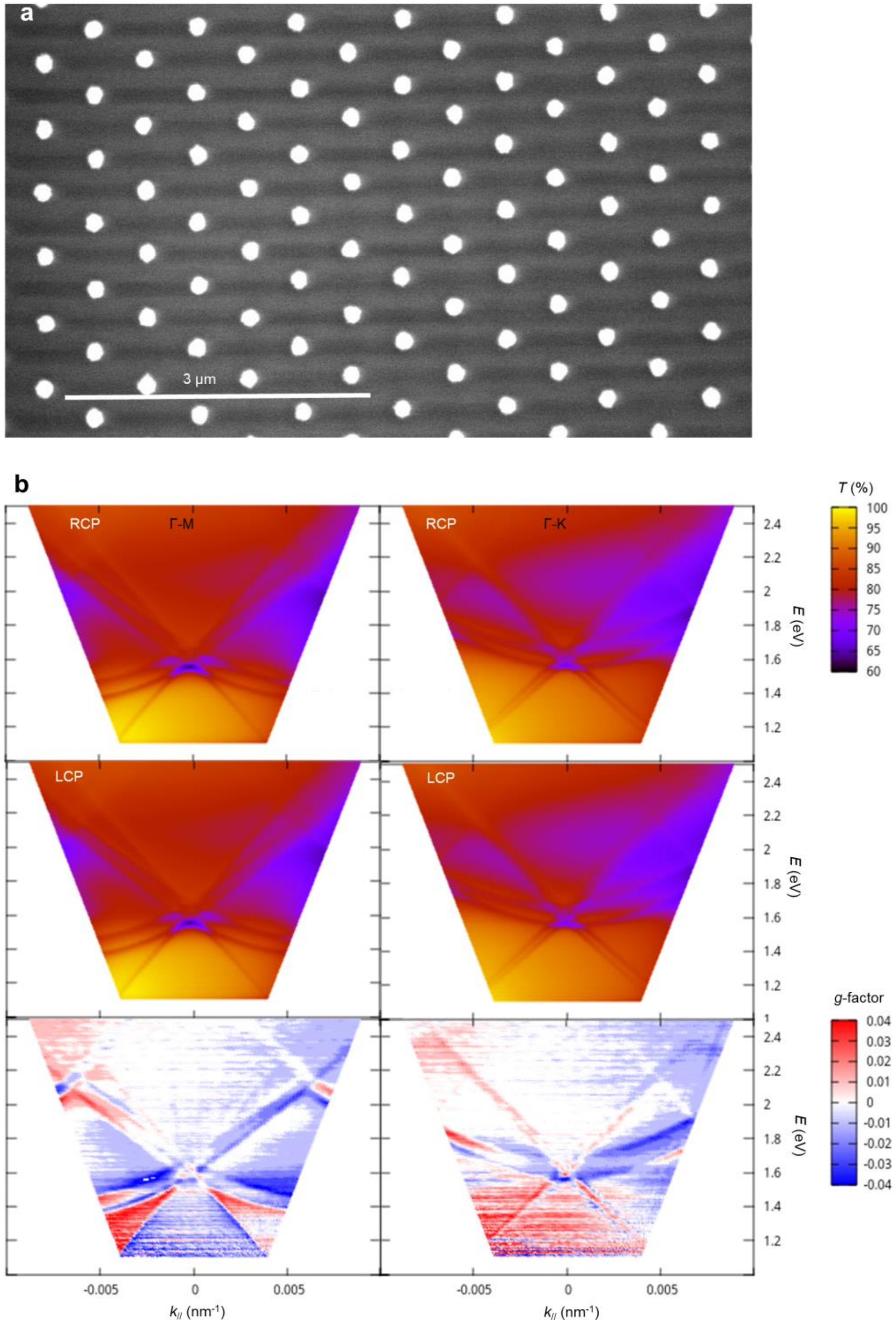

Supplementary Fig. 28. Dichroism in cylindrical hexagonal arrays. SEM image and angle-resolved optical response of 27 nm gold index match (oil) disks in hexagonal arrays. (**a**) SEM image of the disk-like shaped Au nanoparticle clusters in a hexagonal lattice. (**b**) Angular distribution maps in Energy versus wavevector for transmittance under RCP and LCP light in the Γ-M and (**b**) Γ-K directions and its corresponding *g*-factor maps.

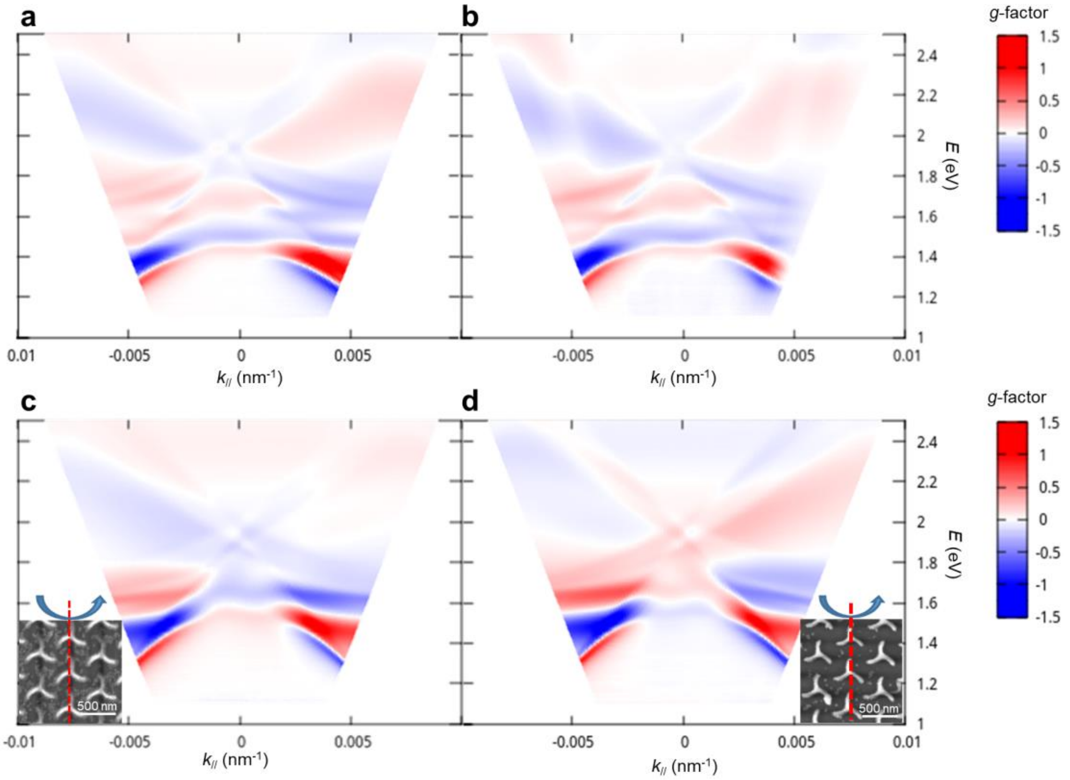

Supplementary Fig. 29. Angle-resolved optical response. Response of 32 nm (**a**, **b**) and 27 nm (**c**, **d**) silver nanoparticle triskelia arrays with index match oil for different azimuthal orientations. (**a**, **b**) forward and backward incident light direction. (**c**) R- and (**d**) L- triskelia arrays at equivalent azimuthal orientation. The SEM in the inset depict the rotation axis (red dashed line). Color bar scale corresponds to *g*-factor.

Evolution of the *g*-factor angular dispersion of the arrays with the azimuthal angle.

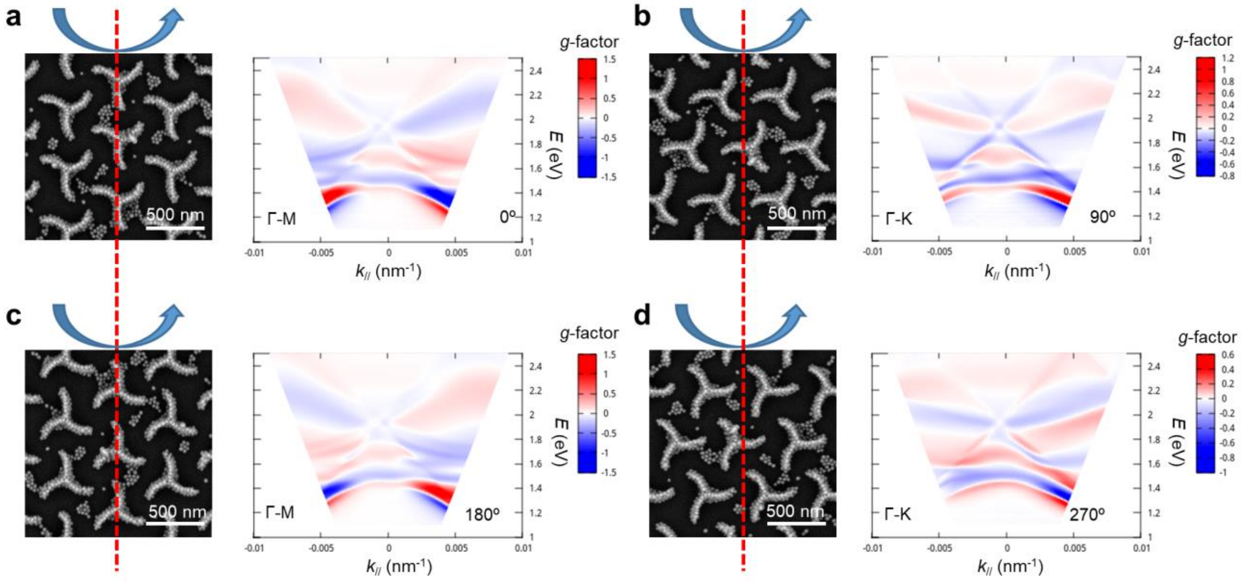

Supplementary Fig. 30. SEM images and angle-resolved optical characterization. Response of 32 nm silver R-triskelia arrays with index match oil for different azimuthal orientations (the red dashed line in each SEM image represent the rotation axis). Color bar corresponds to *g*-factor.

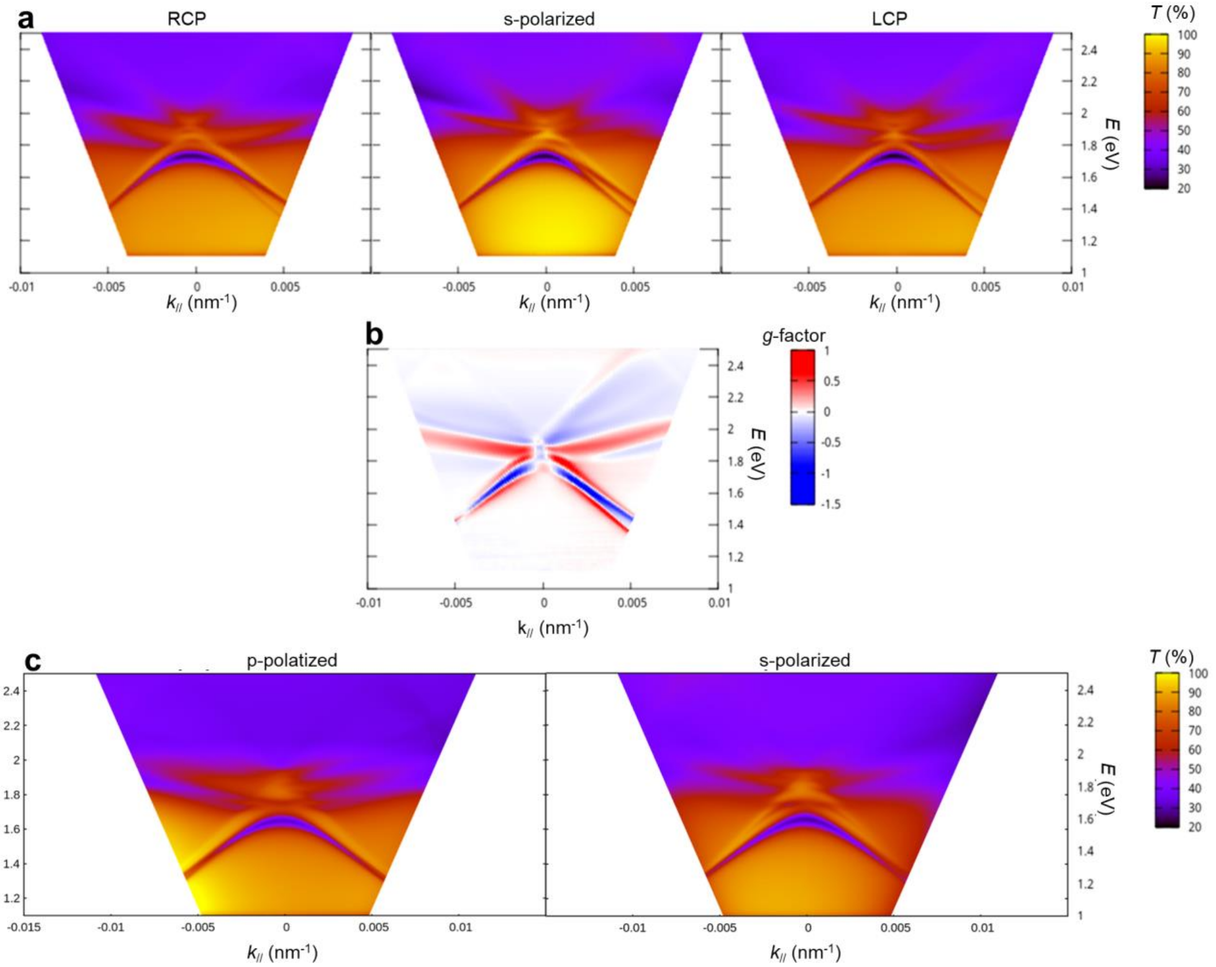

Supplementary Fig. 31. Angle-resolved optical characterization. Response of 27 nm silver triskelia arrays with index match oil. (**a**) Transmittance angular dispersion collected under LCP, s-polarized and RCP. (Γ-K) (**b**) Angular distribution of the *g*-factor. (**c**) Transmittance angular dispersion collected under p- and s-polarized. (Energy versus wavevector *k*).

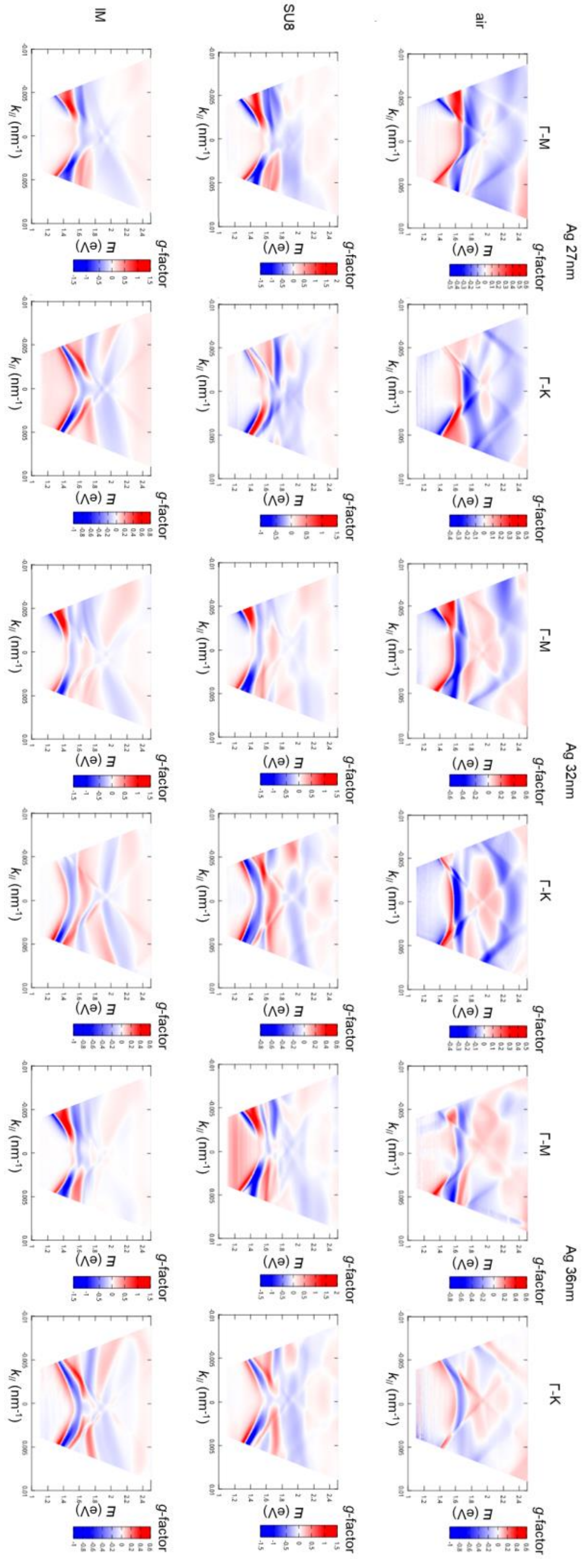

Supplementary Fig. 32. Angle-resolved *g*-factor response (Energy versus wavevector *k*) for silver NPs of 27, 32 and 36 nm in R-triskelia arrays. Two rotation directions are presented according to Supplementary Fig. 28 (Γ-M and Γ-K) and three different superstrates: air, SU8 and IM oil.

Suppl. Note 10. Circularly polarized photoluminescence

Maximum *g*-factor values found for Ag triskelion arrays covered with SU8 resist ($n \approx 1.6$) under oblique incidence. These values correspond to those shown in Fig. 5a of the main manuscript.

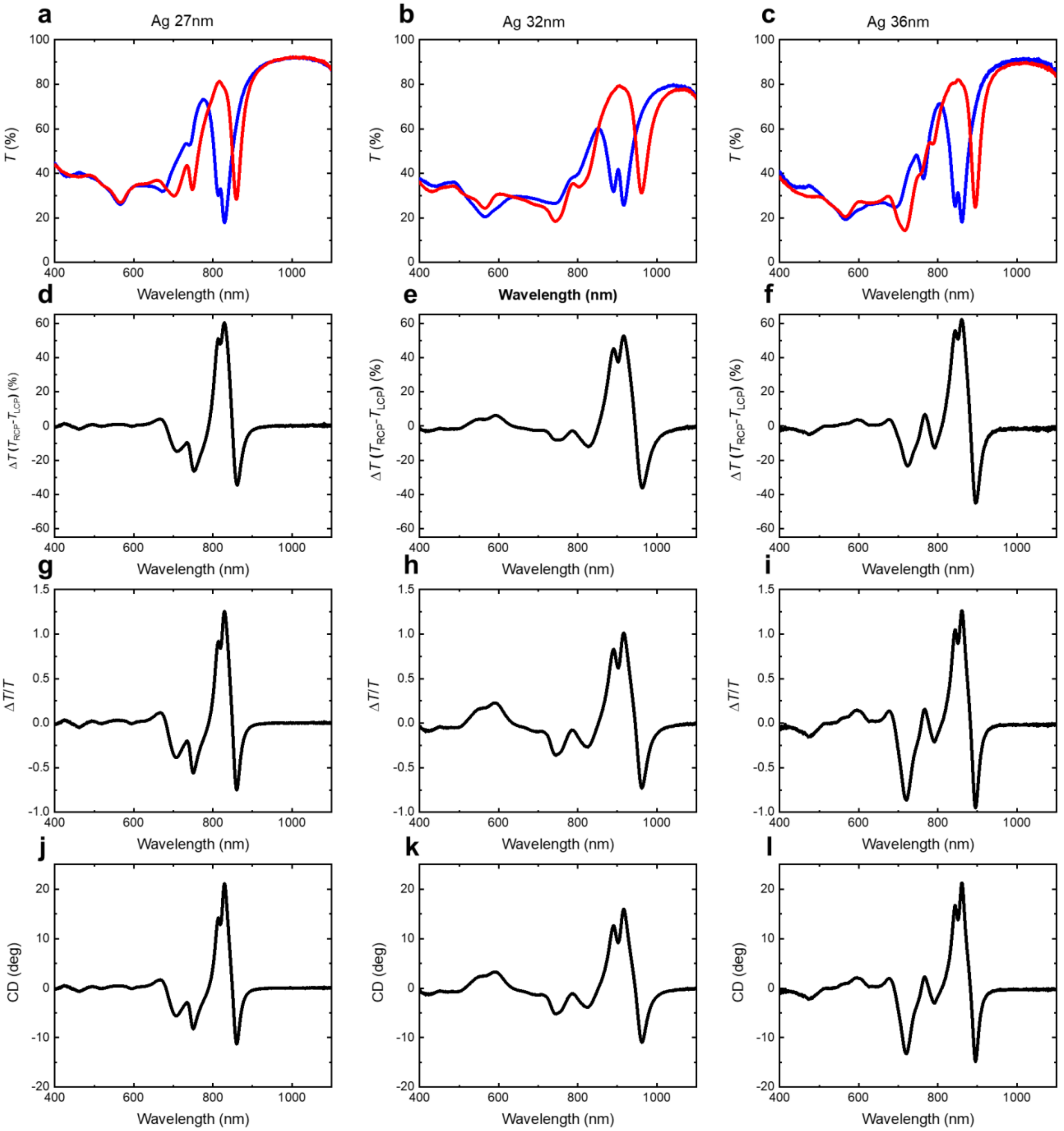

Supplementary Fig. 33. (**a-c**) **Maximum *g*-factor values found for Ag triskelion arrays covered with SU8 resist ($n \approx 1.6$).** Transmittance spectra (RCP, red lines, LCP blue lines), (**d-f**) ΔT, (**g-i**) $\Delta T/T$ and (**j-l**) CD in deg. at oblique incidence (angle of incidence AOI) as shown in Fig. 5a, for silver nanoparticle triskelia arrays. (**a**, **d**, **g**, **j**) 27 nm (AOI = 20º), (**b**, **e**, **h**, **k**) 32 nm (AOI = 20º) and (**c**, **f**, **i**, **l**) 36 nm (AOI = 30º) with SU8 coating.

A solution of SU8 at 7 wt.% photoresist doped with RhB or IR-140 was prepared as emitting index matching layer. The solution is spin-coated at 2000 rpm to ensure good uniformity of the layer over the entire substrate (300 nm thick film).

Images of the colored layers onto the samples after the spin coating are shown in Supplementary Figs. 34a, b. A green laser (λ_{ex} = 532 nm) was used to excite the PL in samples coated with RhB doped resist. The samples covered with the IR-140 doped resist were excited with λ_{ex} = 760 nm from a tunable white laser.

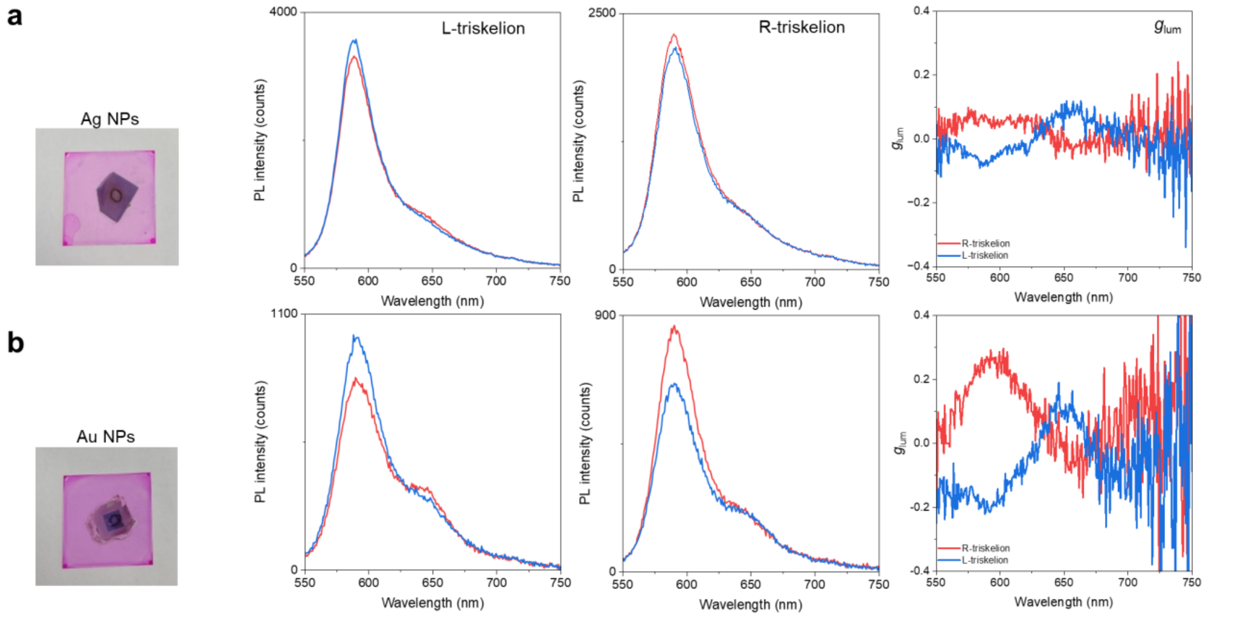

Supplementary Fig. 34. CPL for triskelion arrays coated with RhB at normal incidence. CPL observed from the (**a**) Ag 32 nm and (**b**) Au 45 nm NPs triskelion arrays coated with RhB doped resist.

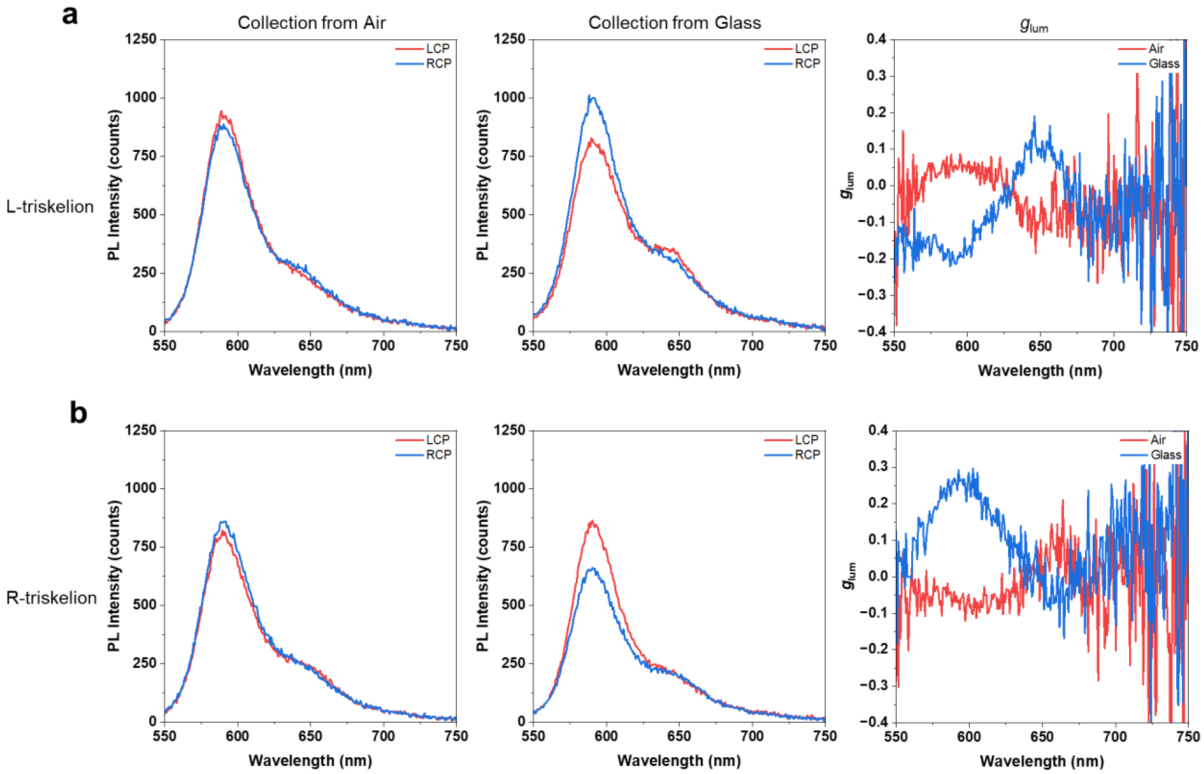

Supplementary Fig. 35. CPL for triskelion arrays coated with RhB at normal incidence. RhB Circularly Polarized Photoluminescence observed from (**a**) L- and (**b**) R- triskelion arrays (Au 45 nm NPs). Light detection though the glass substrate or in the reverse direction.

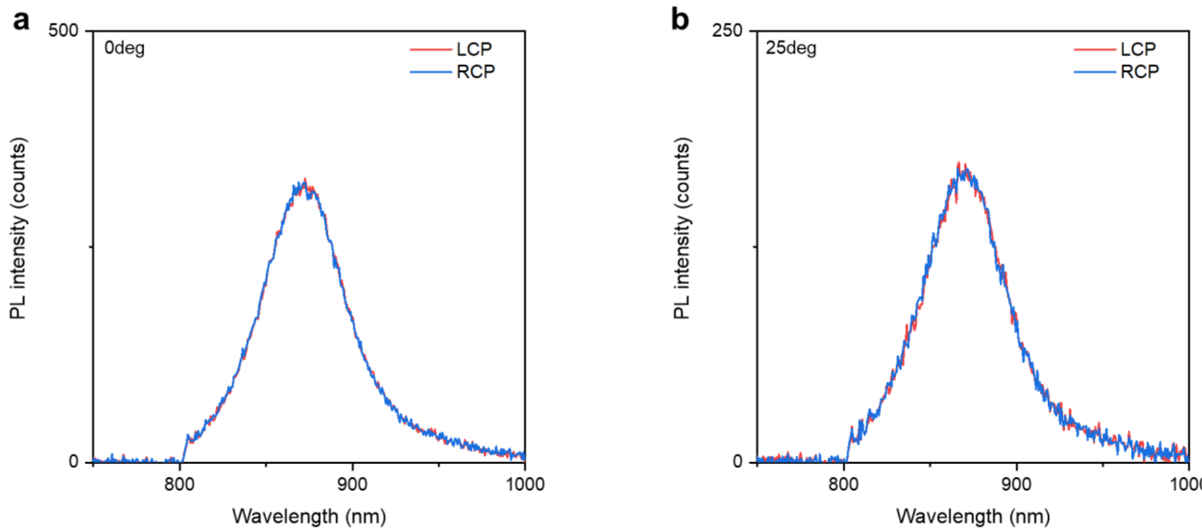

Supplementary Fig. 36. **IR-140 CPL observed OUTSIDE of the triskelion patterns under normal incidence.** (**a**) and at oblique incidence 25º (**b**), red line LCP and blue line RCP.

Another characteristic of the extrinsic chirality in plasmonic lattices is that, as the SLR, spectral position depends on the AOI, the *g*-factors corresponding to the differential coupling to the SLR also shift. Therefore, when circular polarized light emission measurements were conducted for different angles of incidence on Ag NP triskelion arrays, as shown in Supplementary Figs. 37a-e,

for 0º, -10º, -20º, -25º, and -30º, respectively, the g_{lum} features calculated for each angle, such as shape and position of the maximum, depict a marked dependence with the AOI. At normal incidence (0º), there is a slight increase of the g_{lum} at the lower limit of the studied range. When the AOI is -10º, a g_{lum} = 0.3 is observed at 807 nm. At -20º, the maximum g_{lum} shifts to 835 nm, while at -25º, its magnitude increases to 0.37 at 850 nm. Another notable characteristic is that at λ < 825 nm, the observed g_{lum} values change sign. Finally, at -30º, the maximum is located near 874 nm.

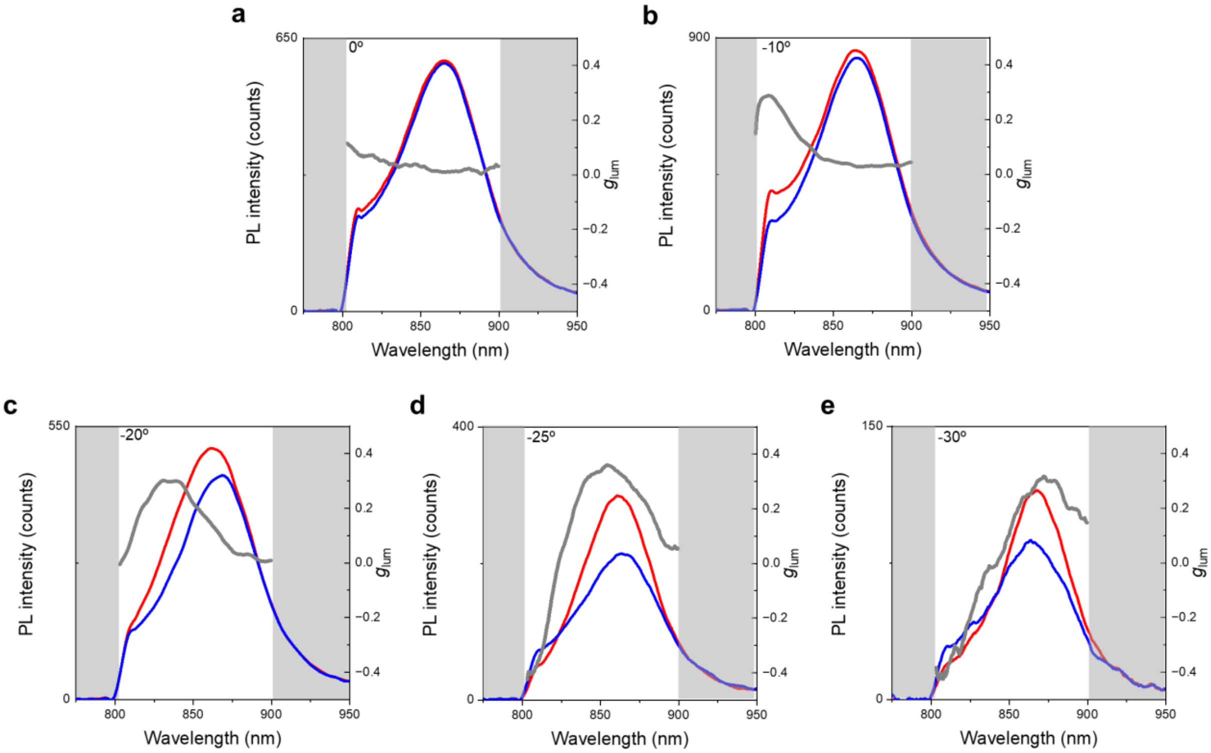

Supplementary Fig. 37. CPL and calculated g_{lum} from silver triskelion array on top of a high index substrate (*n* = 1.6) coated with IR-140 doped SU8 resist (*n* = 1.6) at different AOI. (**a**) 0°, (**b**) -10°, (**c**) -20°, (**d**) -25° and (**e**) -30º. LCP (red lines), RCP (blue lines).
